# Links between physical and chemical weathering inferred from a 65-m-deep borehole through Earth’s critical zone

**DOI:** 10.1038/s41598-019-40819-9

**Published:** 2019-03-14

**Authors:** W. Steven Holbrook, Virginia Marcon, Allan R. Bacon, Susan L. Brantley, Bradley J. Carr, Brady A. Flinchum, Daniel D. Richter, Clifford S. Riebe

**Affiliations:** 1 Department of Geology and Geophysics, University of Wyoming, Laramie, WY 82071 USA; 2 Department of Geosciences, Virginia Tech, Blacksburg, VA 24061 USA; 3 Department of Geosciences, Pennsylvania State University, University Park, PA 16802 USA; 4 Soil and Water Sciences Department, University of Florida, Gainesville, Florida 32611 USA; 5 Nicholas School of the Environment, Duke University, Durham, NC 27708 USA

## Abstract

As bedrock weathers to regolith – defined here as weathered rock, saprolite, and soil – porosity grows, guides fluid flow, and liberates nutrients from minerals. Though vital to terrestrial life, the processes that transform bedrock into soil are poorly understood, especially in deep regolith, where direct observations are difficult. A 65-m-deep borehole in the Calhoun Critical Zone Observatory, South Carolina, provides unusual access to a complete weathering profile in an Appalachian granitoid. Co-located geophysical and geochemical datasets in the borehole show a remarkably consistent picture of linked chemical and physical weathering processes, acting over a 38-m-thick regolith divided into three layers: soil; porous, highly weathered saprolite; and weathered, fractured bedrock. The data document that major minerals (plagioclase and biotite) commence to weather at 38 m depth, 20 m below the base of saprolite, in deep, weathered rock where physical, chemical and optical properties abruptly change. The transition from saprolite to weathered bedrock is more gradational, over a depth range of 11–18 m. Chemical weathering increases steadily upward in the weathered bedrock, with intervals of more intense weathering along fractures, documenting the combined influence of time, reactive fluid transport, and the opening of fractures as rock is exhumed and transformed near Earth’s surface.

## Introduction

The “critical zone” (CZ) is Earth’s breathing skin–the surface layer spanning treetops to groundwater across which key physical and biogeochemical processes transport energy, water, carbon and nutrients^1^. Fundamental to the CZ is the transformation of intact bedrock (protolith) into regolith –the near-surface layer of weathered bedrock, saprolite, and soil. Regolith provides much of the habitable substrate for Earth’s terrestrial life^2^. By producing porosity and releasing nutrients used by ecosystems, weathering creates soils that support ecosystems and human societies^3^. Weathering is also central to landscape evolution, as it prepares rock for transport by erosion and mass wasting at the surface^4,5^. The formation of regolith is therefore fundamental to a wide range of disciplines, including watershed hydrology, geomorphology, soil science, and ecology^6^.

Because the deep CZ is hidden from view, its thickness, structure, variability, and controlling factors remain poorly understood. Recent hypotheses for regolith evolution emphasize chemical processes such as reactive transport^7^, geomorphological processes such as erosion and channel incision^8^, hydrological processes such as groundwater flow^9,10^, and physical processes such as frost cracking^11^ and topographic and tectonic stresses^12,13^. These hypotheses likely act in concert in some locations and independently or in competition in others^2^. Progress in understanding the relative roles of, and controls on, these processes requires improved knowledge of CZ structure across multiple scales, from mineral grains to landscapes. A key but thus far largely untested question is whether chemical and physical indicators of weathering agree, especially in the deep CZ. The base of regolith can be defined in terms of chemical reactions and equilibrium between pore waters and rock^7,14^, in terms of access and drainage of meteoric water^8^, or in terms of changes in physical properties at depth that appear to be connected to the surface^13^. Are these boundaries one and the same?

Models of regolith development generally predict that the base of regolith, while deeper beneath ridges and shallower beneath drainages, shows a convex-upward shape that mimics topography near ridgetops^7,8^. Recent geophysical images in several areas, however, including slopes near our study site, show “bow-tie” shapes in which the interpreted base of weathered bedrock is concave-upward, forming a mirror image of surface topography similar to images of hydrologic flowpaths below hills^15^ — shapes that may express topographic stress patterns in areas of regional compression^13^. The geophysical images have been interpreted as indicating an important boundary in the deep CZ that corresponds to P-wave velocities of about 4–4.5 km/s in crystalline rock^16^. Until now, however, no data have explored whether this deep boundary also corresponds to the onset of chemical weathering.

Here we present results of a combined geophysical and geochemical study of the deep CZ in the Calhoun Critical Zone Observatory (CZO) in South Carolina. Our work focuses on a 65-m-deep borehole that provides unusual access to the physical and chemical properties of the deep CZ^17^. Surface geophysics, downhole geophysical logging, and geochemical data show that reduced seismic refraction velocities coincide with the weathering fronts of major minerals, including plagioclase and biotite. The data can be explained by topographic and tectonic stresses allowing fractures to open at the same time that non-equilibrated meteoric waters access and weather the deep subsurface, either along fractures and/or through matrix flow, likely driving mineral reactions that contribute to fracturing. Our results show remarkable correspondence between physical and chemical properties deep in the CZ. In addition to this unique dataset, we provide interpretations and hypotheses related to how physical fracturing, chemical weathering, and lithology interact in initiating weathering in the deep CZ.

## Geologic Setting and Methods

The well site lies in the Appalachian Piedmont province, 15 km southwest of Union, South Carolina. Bedrock forms part of the Cat Square terrane, a complex of meta-igneous rocks of Neoproterozoic to Cambrian age^18^. The protolith is a granitic gneiss with a weak foliation and some intervals of more abundant light-colored minerals such as quartz. Above 55 m depth, physical and chemical variations in the rock are slight and can be interpreted with respect to weathering, as described below. Beneath ~55 m, in contrast, although there is little evidence for substantial bulk weathering (low porosity and high sonic velocity), the rock appears to have a significantly different composition, with more felsic quartz-rich zones, a higher Fe content, highly variable color, and measurably different electrical resistivity (Supplementary Information, “Lithology below 55 m”). We therefore used the composition and microscopic imaging of samples from 41 m to 53 m to describe a mineral model for the average estimated granitic protolith: ~38% plagioclase, ~28% quartz, 22% orthoclase, 4% biotite, 3% epidote, and minor amounts of calcite (~0.1%) and pyrite (~0.01%) (Supplementary Information, “Geochemical Analyses and Calculations”).

The Calhoun CZO has a humid subtropical climate, with mean annual precipitation of ~1270 mm and mean annual temperature of ~16 °C. Although the well site is on a broad, relatively flat interfluve (~200 m elevation above sea level), the surrounding landscape is typified by gentle to moderately steep hillslopes, with erosional gullying dating from farming practices in the 19^th^ and early 20^th^ centuries. Relief to the base of nearest streams is ~25 m. Soils at the site are highly weathered Ultisols (fine, kaolinitic, thermic soils in the family Oxyaquic Kanhapludult). Minimum residence time for the soil and regolith is 2 to 3 million years, based on meteoric ^10^Be data^17^. Vegetation consists of forests of loblolly pine, shortleaf pine, and hardwoods^19^, including hickory and oak^17^.

We acquired geophysical data, both on the surface at the site and using downhole logging tools. To document chemical changes in the rock, we analyzed bulk geochemistry from cuttings and cores recovered during drilling and used mass balance principles to quantify mass transfer coefficients of individual elements (*τ*). Geophysical measurements included surface seismic refraction data and downhole measurements of acoustic, optical and chemical properties of the wall rock (Supplementary Information, “Data Acquisition”). Porosity was measured from borehole nuclear magnetic resonance (NMR) measurements and estimated from a petrophysical model applied to compressional-wave borehole sonic velocities (Supplementary Information, “Rock Physics Modeling”).

## Results

**Figure 1:**
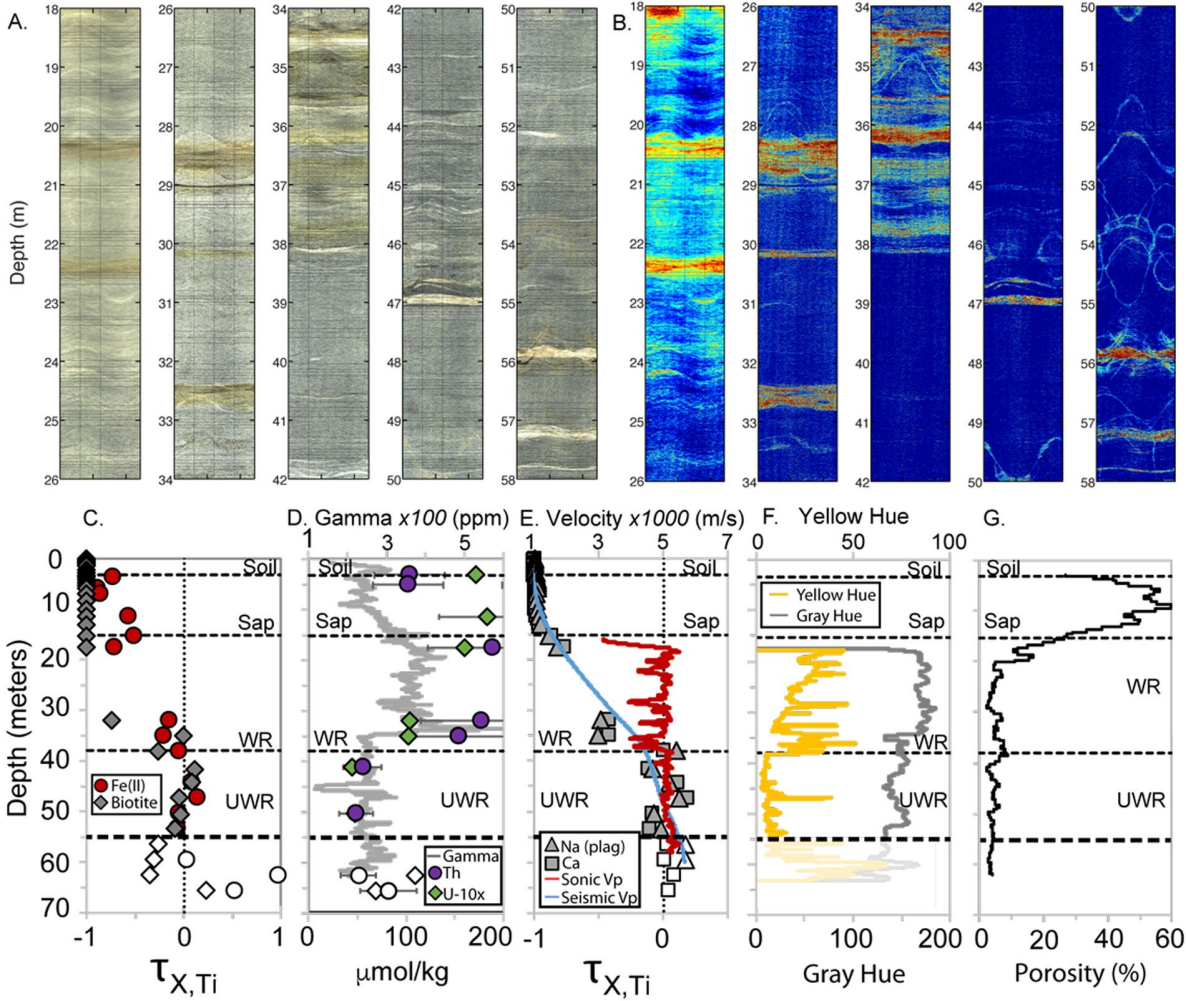
Downhole borehole images, geochemistry, and geophysics. Optical borehole imager view of the borehole wall (**A**) and yellow hue, defined as (R + G)/2-B (**B**). Inclined planar features such as fractures appear as sinusoids on these unrolled 360° images of the borehole wall. Warm colors in (B represent higher levels of yellow and brown; cooler colors represent gray. Above 55 m depth, yellow/brown versus gray hues generally indicate weathered versus unweathered minerals, respectively; below 55 m, a different rock exists and yellow colors do not necessarily indicate weathering. Downhole geochemical and geophysical data demarcate the onset of weathering around 38 m depth (**C**–**G**), which corresponds with visual changes in A and B. Interpreted layers in **C–G** are soil, saprolite (Sap), weathered rock (WR) and unweathered rock or protolith (UWR). Oxidation of the rock is represented by a loss of Fe(II) and alteration of biotite (**C**). Changes in U, Th and natural gamma (**D**) demarcate a possible lithological change at 34 m. Slowing seismic and sonic velocities and loss of plagioclase feldspar (Plag), Na and Ca (**E**) are consistent with a boundary between unweathered and weathered rock around 38 m. Refraction seismic velocity is derived from two lines that cross the borehole^13^. Fractional mass loss (X = Fe(II), biotite, or plagioclase), τ_X,Ti_, was calculated assuming Ti is immobile during weathering. τ varies from 0 (no mass loss relative to Ti in protolith) to −1 (100% loss). Open symbols on (**C**,**D** and **E**) represent a different lithology below the protolith at 55 m. Below 34 m, the rock is darker (lower grayscale value, **F**), and less radioactive (natural gamma, **D**). Measures of borehole wall color with depth (**F**) (gray hue = gray line; yellow hue = yellow line) show upward increasing yellow hue indicative of weathering (as observed in **B**). Grayscale and yellow hue are plotted as faint lines below 55 m, where we interpret a different lithology unrelated to the shallow critical zone. Finally, NMR-derived porosity (**G**) increases at the unweathered rock-weathered rock boundary and again throughout the saprolite as seismic velocities decrease and chemical depletion increases.

The borehole provides an uncommonly detailed one-dimensional view of chemical and physical processes that contribute to weathering and porosity production in regolith (Fig. 1). The crystalline bedrock has a low initial porosity (~4% estimated by nuclear magnetic resonance (NMR)), a gray/white coloration that contrasts with yellow/brown weathering stains shallower in the profile, and a relatively uniform felsic lithology with some heterogeneities discussed below. We use the protolith as a background against which changes in physical properties, chemical composition, and color are interpreted. Here we present the principal observations of the subsurface, working upward from the intact bedrock at depth to the soil. From the data described below, we define three layers based on observed changes in the optical, physical, mineralogical, and geochemical properties at 38 m and from 11 to 18 m depth. We argue that detectable weathering commences at 38 m, where geochemical compositions begin to vary markedly from that of the protolith (estimated as an average of samples from 41 to 53 m). The zone between 18 and 38 m is described as weathered and fractured bedrock and the zone between 3 and 18 m as saprolite. The A, E, and B horizons of the soil are between 0 and 3 m.

### Protolith

**Figure 2:**
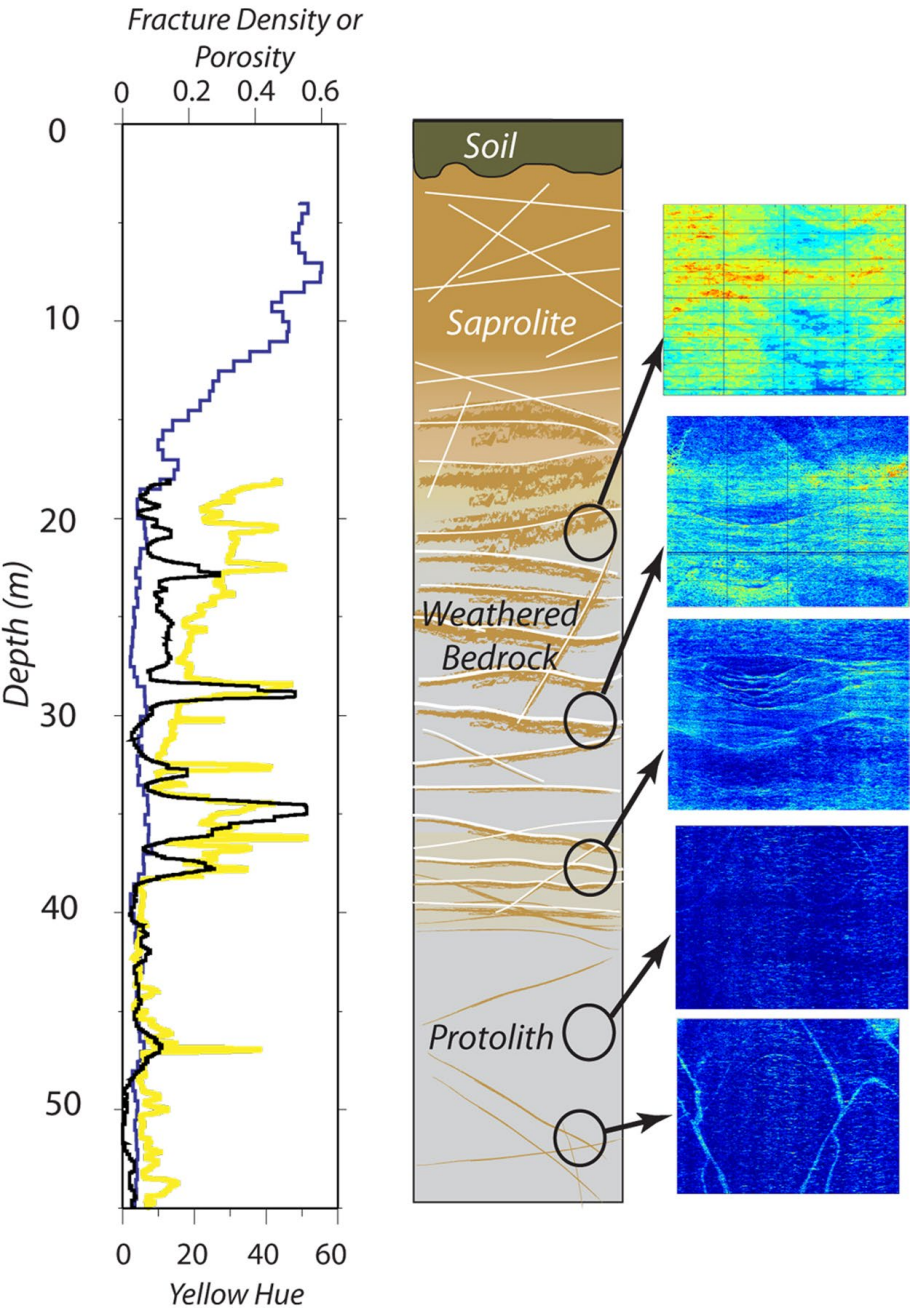
Water and fractures in the critical zone. Depth plots of water content (blue line) from the NMR log (i.e., total porosity below the water table observed at about 4 m, fracture density (black line), and yellow hue (yellow line). Conceptual model of the Calhoun borehole rock (***Center***) shows upward increase in fracture density and weathering (brown color). Zooms of yellow hue from OBI at several levels of the well (***Right***) highlight the association of weathering (warm colors) with fractures (sinusoidal shapes) at all depths, and overall upward increase in weathering intensity from 38 m to 18 m depth.

The rock between ~38 and 53 m depth has relatively invariant geophysical and geochemical properties consistent with largely unweathered rock. Chemical depletion estimates (*τ*) vary around zero in this depth interval, reflecting small variations in parent composition (Fig. 1). Refraction velocities in this zone are high (4–5 km/s), sonic log velocities are high (~5 km/s) and fluctuate little (Fig. 1E), and fracture density is low (~4%) (Fig. 2). Volumetric water contents from the NMR profile indicate that porosity is low (~4%) (Fig. 1). In optical borehole images (OBI), the rock is an overall dark gray color with bands of whiter, presumably quartz-rich veins that may also contain other light-colored minerals (Fig. 1A), some of which appear to have inherent yellow hues unrelated to chemical weathering (Fig. 1B). Staining due to weathering occurs in thin zones along hairline fractures or lithological contacts, as indicated by televiewer images (Fig. 1A,B), optical observations in thin sections, and the calculated yellow hue (Figs 1 and 2). Natural gamma values are relatively constant throughout this zone, with the prominent exception of a negative anomaly centered at 45 m (Fig. 1D). This negative gamma anomaly largely coincides with a zone of slightly darker rock (lower grayscale value) and slightly higher Fe(II) concentrations, consistent with a zone of more mafic minerals.

The values of τ for samples from 38 to 53 m (Supplementary Tables S1 and S2, Fig. 1) show little variation for Si, K, and Na. The values of τ for Al, Ca, and Mg vary more, indicating the variability in protolith composition around our estimated average. This variation is likely not reflective of variations at the scale of the banding observed in Fig. 1A because protolith samples were homogenized over 3 m depth intervals.

### Weathered Bedrock

**Figure 3:**
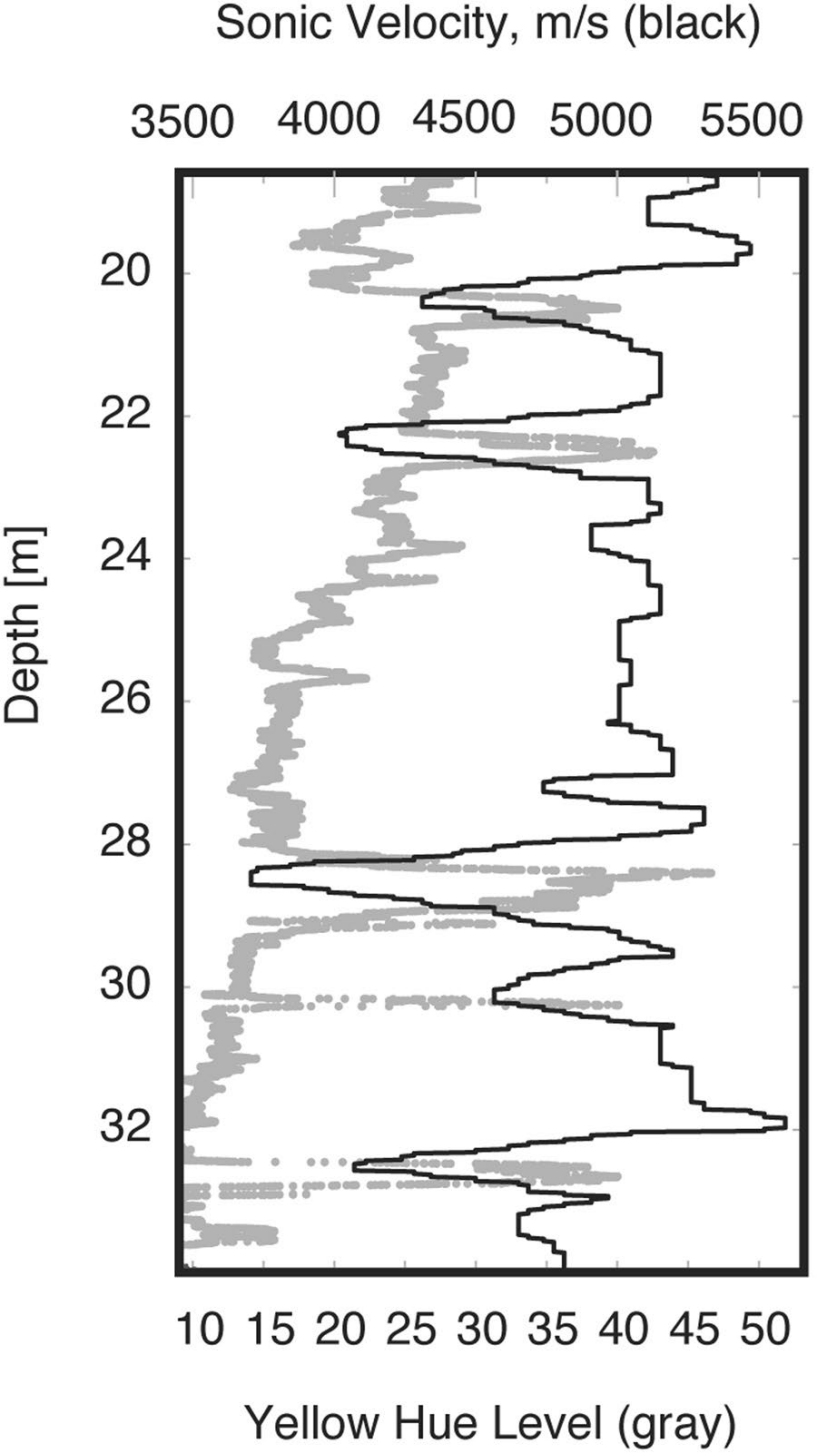
Correlation of yellow hue and sonic log velocity between ~19–34 m depth. Decreases in sonic velocity (black) correspond to increases in yellow hue (gray), which we attribute to the combined effect of chemical weathering and fracturing.

The zone between 18 and 38 m depth, which we interpret as weathered and fractured bedrock, is characterized by rapidly changing optical, physical, and chemical properties. Optically, this zone contains more conspicuous yellow/brown staining than the rock below 38 m (Fig. 1A,B). The yellow hue value of this zone shows a steady upward increase punctuated by spikes of concentrated weathering (Figs 1F, 2 and 3). This upward change in yellow hue does not appear to be a consequence of turbidity in the hole (Supplementary Information, “Fourier Analysis of Optical Borehole Images”). Geophysical properties in the fractured bedrock show clear contrasts to the protolith, with reduced surface refraction velocities (<4 km/s) and variable sonic log velocities that fluctuate between ~3700 and 5500 m/s (Figs 1E and 3). Surface seismic refraction velocities decrease upward from ~4.0 km/s at 38 m depth to 1.4 km/s at 18 m depth (Fig. 1E). Fracture density measured from acoustic borehole imaging (Supplementary Information, “Data Acquisition/Borehole logging”) is high (average ~16%) but variable throughout the layer (black curve, Fig. 2).

**Figure 4:**
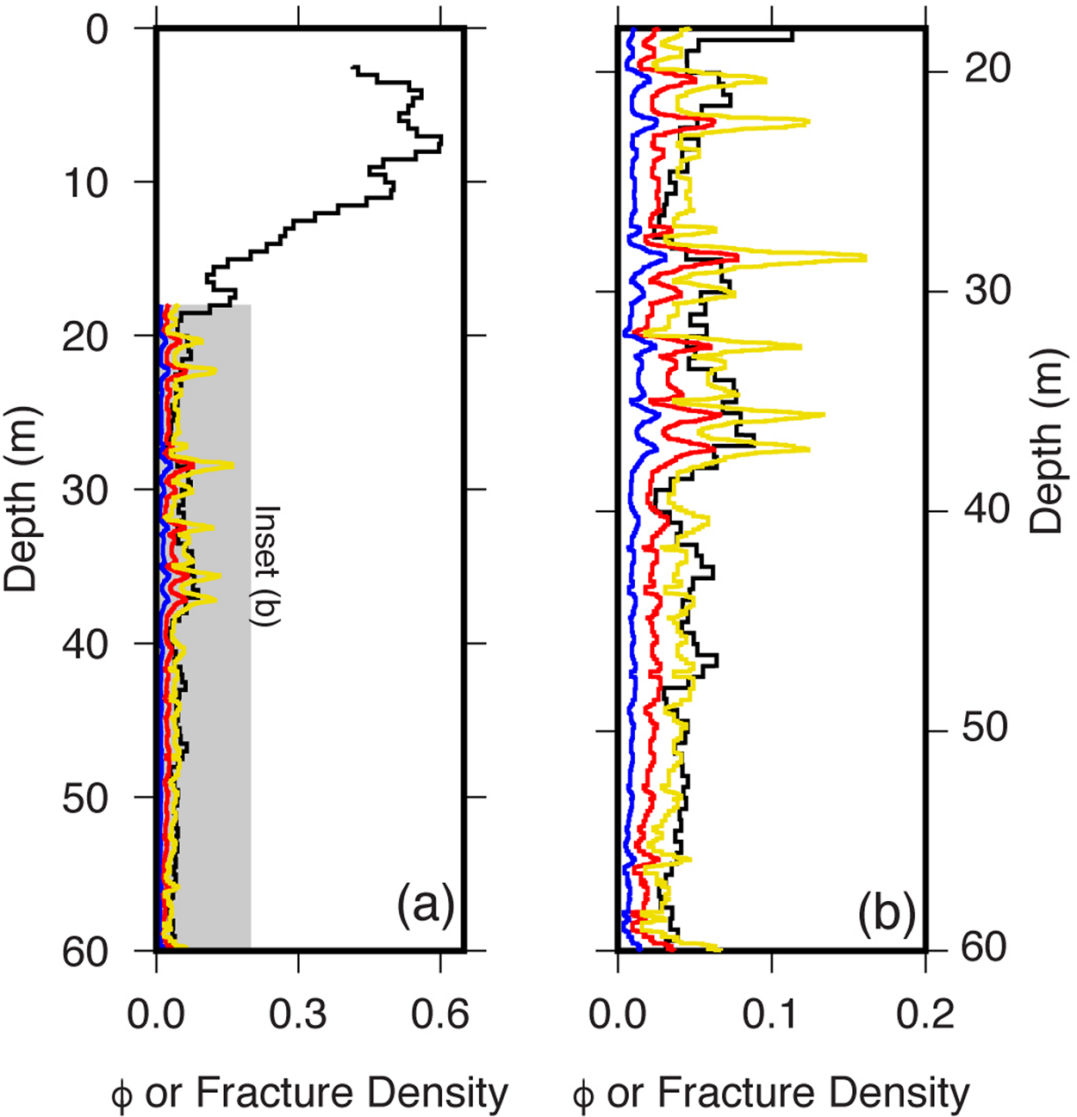
Porosity from geophysics. (**a**) Comparison of NMR-derived porosities from the saturated zone (black) to porosities estimated from sonic logs (colors) under several assumptions: fully saturated fractures (red line), air-filled fractures (blue line), kaolinite-filled fractures (yellow line). Method for calculating fracture density is described in the Supplementary Information under “Rock physics modeling”. (**b**) Zoom of porosities within the uncased region of the borehole, plotted as in (**a**). Porosities from NMR were determined from T2* relaxation times (see Supplementary Information).

Porosities inferred from both NMR and sonic logs in the weathered bedrock layer are relatively low (<10%) but higher than in the protolith. NMR porosities average 5.5% but show some variability that can be petrophysically modeled (Supplementary Information, “Rock Physics Modeling”) as fluid- or clay-filled fractures (i.e. 3.2% or 5.9%, respectively, Figs 2 and 4). Zones of slightly higher porosities in the NMR inversions at 34–38 m (6–9%) and 28–29 m (~7%) correspond to zones of higher fracture density and higher yellow hue values (Figs 1E and 3). Despite these variations, NMR-inferred porosities remain below 10% until the transition toward saprolite at 18 m depth. The 38 m depth represents a transition in NMR porosity: from 37 to 39 m depth, porosity decreases from ~9% to ~2%.

Measurable chemical depletion of some elements relative to the defined protolith occurs in all samples above 38 m depth (τ values become negative in Fig. 1C,E). Most significantly, Ca, Na, K, Al, and Si show significant depletion starting at that depth. By the top of fractured bedrock at 18 m depth, mass losses reach ~70% for Na and Ca and ~30% for K, Al, and Si.

A change in properties at 34 m depth bears special mention. This depth corresponds to a marked change in grayscale value; rocks deeper than 34 m are darker than those above that depth (Fig. 1F). Similarly, a sharp change in natural gamma activity occurs at that depth, with rock below 34 m lower in gamma activity than that in the overlying fractured bedrock (Fig. 1D). Finally, the zone between 34–38 m shows high values of yellow/brown hue (Fig. 1F), clearly observable as staining on the optical images (Fig. 1A,B). Somewhat consistent with these geophysical observations, sharp changes are also observed in U, Th, and Fe(II) contents. Specifically, U and Th increase above 32 m and *τ*_*Fe(II)*_ becomes more negative above 38 m, close to the depth where the natural gamma increases (Fig. 1C,D). One possible interpretation is that there is a slight compositional change in the parent rock at 34 m, from a light gray granitoid to a slightly darker granitoid, though without core samples, we lack definitive data to test this. This possible compositional change may have implications for the degree of inferred chemical weathering in the deep fractured bedrock layer, as discussed further in the Discussion.

### Saprolite

The saprolite layer above 18 m depth has markedly different physical and chemical properties from the underlying weathered bedrock. We lack useful optical borehole images and sonic velocities in the saprolite layer, due to the PVC casing that extends down to 18 m. However, the surface seismic velocities, NMR log, and geochemical data show that the zone above 18 m consists of highly chemically altered and increasingly porous material. Seismic refraction velocities are low in the saprolite layer, ranging from ~0.6 km/s at 3 m depth to ~1.4 km/s at 18 m depth. Porosities inferred from the NMR log are high, reaching 50% at 10 m depth and nearly 60% at 8 m depth (Fig. 4). NMR porosities are not shown above the water table at about 2 m, because NMR-inferred water contents do not represent total porosity in the unsaturated zone.

The transition from saprolite to weathered bedrock begins around 18 m, where significant changes in composition begin. The geochemical indicators of mass loss increase progressively upward from 18 m to 11 m depth, where Ca and Na become almost 100% depleted (*τ*~−1.0) (Fig. 1E), consistent with complete alteration of the most abundant mineral, plagioclase feldspar. The mineral model is also consistent with nearly completely alteration of biotite to secondary minerals above 11 m (Fig. 1).

## Discussion

**Figure 5:**
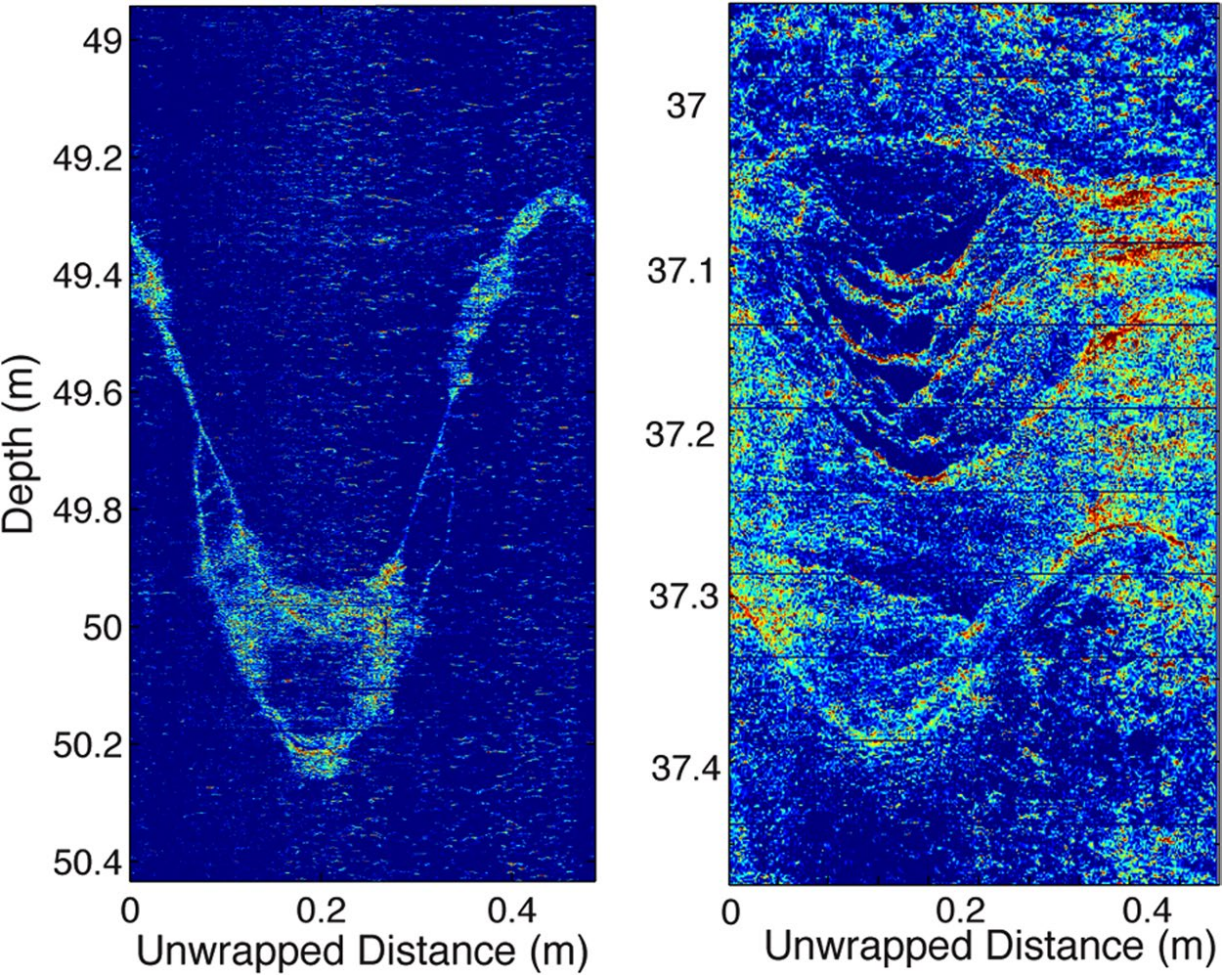
Correlations of fracturing and chemical weathering. Representative zoomed images showing yellow hues associated with intersecting planar fractures (sinusoidal patterns, left) and non-planar fractures (right).

A key observation from the borehole is that chemical weathering and fracturing are co-located. The larger fractures are planar, since they appear as sinusoidal features in unwrapped borehole images. The yellowish hues are interpreted here to document oxidation of ferrous iron in primary minerals, as seen in many rocks^20^; we find no evidence in the samples for significant organic material or other species that could explain the yellow coloration. Weathering inferred from yellow hue in OBI data is strongly associated with fractures at all depths (Figs 1 and 3). The pattern of staining around fractures changes at 38 m coincident with loss of Fe(II) in the rock. Above that depth, staining extends away from fractures well into the surrounding rock, producing thick bands of visually identifiable weathered rock (Fig. 5). Below 38 m, weathering is restricted to very thin bands immediately around fine fractures. At shallower levels, staining is evident throughout the rock material, although it still appears to be most intense immediately adjacent to fractures. All of these observations are consistent with fracture-focused oxidation at depth transitioning to pervasive oxidation in shallower zones.

**Figure 6:**
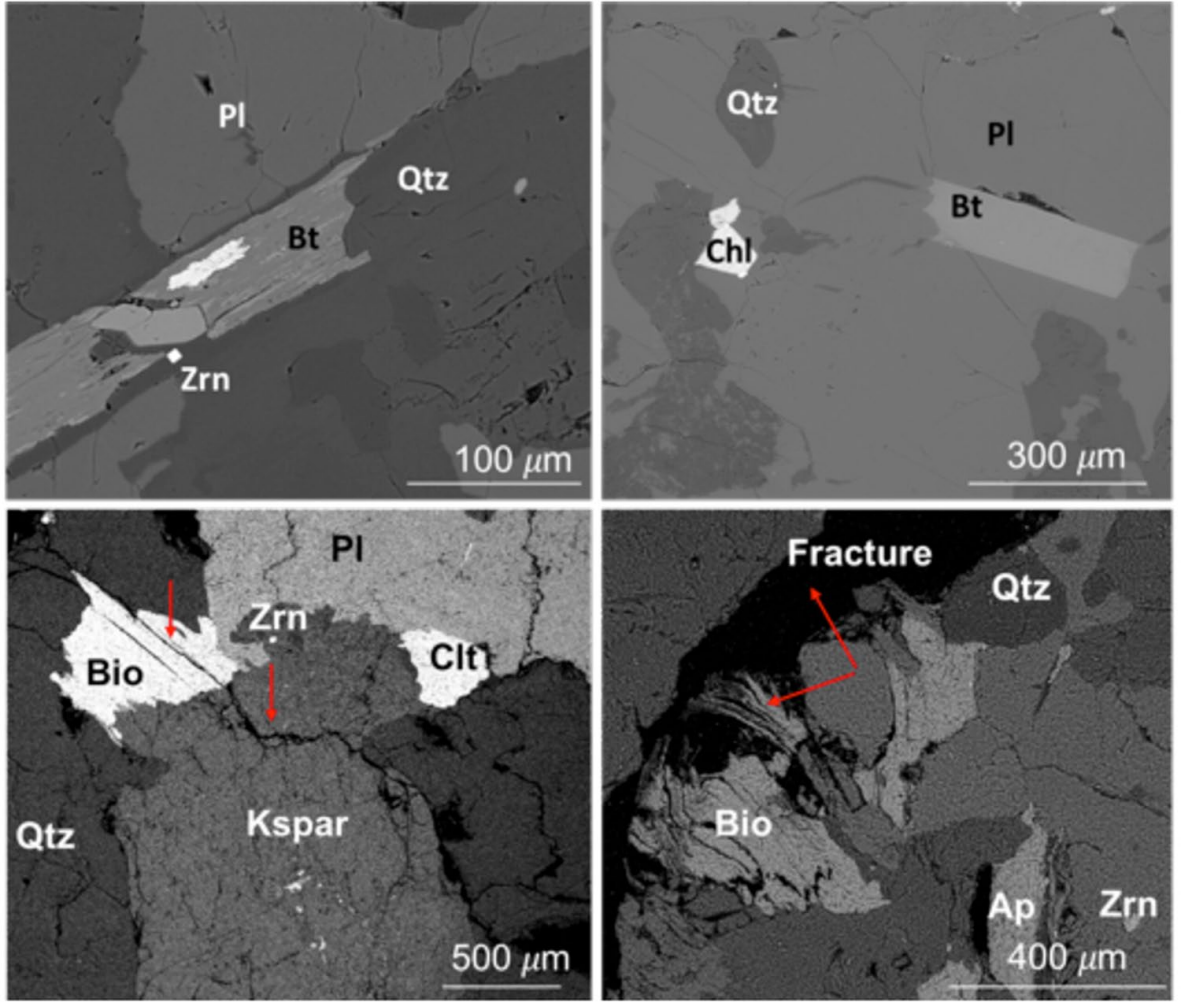
Weathering-induced micro-fracturing. As biotite weathers, Fe(II) is oxidized to Fe(III), causing expansion and weathering-induced fractures. The top two images show a typical back-scattered electron microscope image of the protolith at 50 m depth with no observed cracking around biotite (Bt). The bottom two images from weathered/fractured bedrock at 32 m depth show oxidized biotite (Bio) that has expanded along cleavage planes (dark black parallel lines, marked by red arrow, inside Bio grain) and cracking in neighboring grains (red arrow in potassium feldspar (Kspar) grain). The expansion of biotite is sometimes accommodated in the rock by larger microfractures as shown in bottom right image. These microfractures open the rock to meteoric fluids and enhance weathering. (Labels: Clt or Chl = chlorite; Qtz = quartz; Pl = plagioclase; Ap = apatite; Zrn = zircon).

The patterns of fracturing observed in the borehole images show both planar and non-planar fracture shapes, usually in close proximity. As rock is exhumed to shallower depths, it is pre-conditioned with fractures inherited from tectonic activity deep in the crust^21^ as well as thermoelastic relaxation^22^. Fractures can also be generated or enhanced near the surface by topographic stresses^12,13^ or by expansion during oxidation of biotite or other ferrous iron silicates^23–26^. These processes may result in a range of fractures from meter-scale macrofractures to grain-scale microcracks^22^. For example, tectonic forcing and stress release are expected to produce mostly planar macrofractures. In contrast, mineral expansion is expected to produce grain-scale fracturing that may also coalesce into the meter-scale fracturing that is known as spheroidal weathering^23,27,28^. Observations of thin sections at both 32 and 35 m depth reveal mineral-expansion fractures that propagate outward from weathered biotite (Fig. 6).

Planar and nonplanar macrofracturing would produce contrasting patterns on unwrapped borehole images: inclined, planar macrofractures that cross the borehole produce sinusoidal patterns (e.g., Fig. 5), while nonplanar features such as spheroidal fractures would produce closed circles or distorted sinusoids, depending on the radius of curvature and the point of intersection with the borehole. Fractures in the OBI images are dominantly planar, with a low dip (<20°; Figure S2). This is more consistent with inherited tectonic fractures and/or topographic-stress-related fractures. However, numerous non-planar fractures are also observed (Fig. 5).

The depth interval of high-density fracturing (i.e. down to 38 m) is consistent with the predictions of the topographic stress model for interfluves at nearby sites in the Calhoun CZO^13^, which also reveal refraction velocities >4 km/s at ~40 m depth. However, the close association of planar and non-planar fractures suggests that more than one fracture mechanism is active. Apparently, several types of pathways for meteoric water infiltration are present during weathering at this site. We speculate that stress-related fractures — either inherited from previous tectonics and/or opened and expanded during exhumation through the ambient stress field — provide pathways for meteoric water to infiltrate, thus promoting chemical weathering reactions that enhance mineral-expansion fractures, which may in places coalesce into non-planar macrofractures as observed in our borehole images (Fig. 5).

A striking feature is the large increase in fracturing of different types at the same depth (~38 m) where oxidation and plagioclase weathering are first detected. Oxidation of granitoid rocks often commences with oxidative dissolution of pyrite or biotite^27,29^. No pyrite but abundant biotite was observed in the six thin sections cut from borehole samples. Consistent with the low concentrations of measured sulfur in these samples, we therefore attribute most of the Fe(II) to biotite (~4 weight %), which likely accounts for most of the oxidation that begins at and continues above 38 m. Oxidation of biotite has been associated with expansion of biotite layers and used to explain the development of micro-fractures during weathering of granitic rock^23,26,28,30^. Indeed, thin sections reveal expanded biotite at 32 and 35 m depth (Fig. 6). The initiation of both biotite oxidation and plagioclase dissolution at the same depth (i.e, 38 m (Fig. 1E)) has been observed at other locations^28^. These changes have been attributed to initiation of chemical weathering by oxygen transport into zones containing biotite, followed by oxidation and expansion of the biotite, followed by influx of carbonic acid-charged meteoric fluids, and culminating in plagioclase dissolution^31^.

We attribute the contrast between surface seismic refraction P-velocities (1.4–4 km/s) and sonic velocities (4–5 km/s) in the weathered/fractured bedrock layer (Fig. 1E) to variations in the intensity and scale of fracturing. At the top of the fractured bedrock layer, sonic and refraction velocities differ by a factor of 3–4, but the discrepancy shrinks with depth, such that the two velocities agree at the top of the protolith. Crucially, the discrepancy is not an indication of errors in either seismic or sonic velocities, which are far smaller than the difference between the two velocities. Rather, the discrepancy is a consequence of the differing frequency content of the refraction and sonic methods: seismic velocity tends to be faster when measured at shorter wavelengths (higher frequencies)^32^. In the fractured bedrock, the sonic logs (15 KHz, 5 km/s) have a dominant wavelength of ~0.3 m, while the surface seismic refraction data (40 Hz, 2 km/s) have a dominant wavelength of ~50 m. We attribute the discrepancy between refraction and sonic velocities to the presence of a fracture network at the scale of 10’s of m, which reduces the elastic moduli of the medium at the scale of refraction wavelengths. This could be accomplished by the opening of pre-existing or new fractures in the near-surface stress field^13^, as well as coalescence of fractures by mineral expansion during weathering. We hypothesize that these inferred fractures are the same as those observed in the borehole as prominent weathered zones (e.g., at 22 and 28.5 m depth in Fig. 3). At depths greater than ∼40 m, the agreement of the seismic refraction and sonic velocities suggests that the large-scale fractures are closed or absent, consistent with the optical and acoustic borehole images and derived fracture density estimates.

The change in gamma activity and grayscale value at 34 m strongly suggests a chemical change at that depth. This could indicate a change from a more felsic unit (higher grayscale hue value and higher gamma activity) to an underlying, slightly more mafic composition (lower grayscale hue value and lower gamma activity). At the same depth, the degree of weathering (inferred from yellow/brown hue values) also changes, with greater degrees of weathering in the darker rock (Fig. 4). One interpretation is that lithology, at least in part, is controlling weathering, likely by one of two possible mechanisms. First, the darker rock may contain minerals that weather more readily, such as more Ca-rich feldspars^27^. Second, the darker rock appears to have more fractures (Fig. 4). This could either be a cause or a consequence of the enhanced weathering: if the fractures are pre-existing or stress-release fractures, they could be providing pathways for enhanced access of meteoric water; alternatively, if the fractures are caused by mineral expansion during weathering, the fracturing may follow the weathering.

The potential presence of a lithological change at 34 m depth raises the possibility that our assumed protolith (averaged from 40–53 m depth) may not be the parent rock of the entire critical zone at this site. Even if there is a lithological change at 34 m, there remains strong observational evidence that chemical weathering commences at 38 m, in concert with increased physical weathering: (1) the observed staining, captured by the yellow hue profile (Figs 1 and 2), increases between 38 and 34 m, below the possible lithological change; (2) estimated τ values from the sample at 35 m depth are negative relative to the protolith; and (3) the calculated mineral model at 35 m includes altered biotite as well as increased kaolinite and decreased plagioclase relative to deeper samples (Supplementary Information, Table S6). Thus the question is not whether chemical weathering is present in the fractured bedrock layer, but rather how much weathering. It is possible that the τ value calculated at 35 m depth is affected by some admixed material with a different parent rock, since the three-meter sampling window extends up to 33.5 m, a half meter above the potential lithological change.

If there is a lithological change at 34 m depth, then the τ values estimated for samples at and above 32 m could be inaccurate, if their parent rock compositions differed substantially from the rock sampled at 40–53 m depth. Two lines of evidence suggest that the τ values of about −0.40 are not representative of the entire fractured bedrock layer. First, the sonic and NMR logs show that the porosity of the fractured bedrock is about 5–6% (Fig. 4), well below what would be expected for the mass loss implied by tau values of −0.37 to −0.51 for the major elements (Si, Al, Ca, K, and Na; Supplementary Information, Table S3). Second, the loss of major minerals in the calculated mineral model is less than that expected for the predicted high mass losses in major elements. To explore the possible effects of a change in parent rock composition at 34 m depth, we explored an alternative model, assuming a parent rock equivalent to the composition of the sample at 32 m depth (Supplementary Information, “Alternative Calculations of Chemical Weathering”). The alternative model predicts less chemical mass loss at all depths (and even predicts mass gain for Si and Al at some depths), but still shows major depletion (at least 50%) in elements dominantly present in plagioclase (Na and Ca) by 18 m depth. There is also a rapid loss in K and Mg above 4 m depth in both models, likely related to the loss of orthoclase, biotite and clays.

The vertical and lateral distribution of chemical and physical weathering is vitally important to understanding CZ processes and both are difficult to observe. Despite the uncertainties in parent rock composition, the geochemical and borehole geophysical data acquired from the deep borehole at the Calhoun CZO provide fresh perspective on this question, revealing a detailed vertical snapshot of weathering that can be linked to surface geophysical data that offer an extrapolation into the horizontal dimension over hundreds of meters. At the Calhoun CZO, the base of the weathered bedrock layer is marked by a boundary in physical, optical, chemical and mineralogical properties at 38 m depth. We interpret this depth as the base of significant chemical weathering at this site, but note that some mineral weathering does occur at depths greater than 38 m (Figs 1, 2 and 6), notably along hairline fractures in the rock, documenting influx of oxidizing fluids even at these deeper depths. But these zones are thin and, as shown by the τ values and sonic log velocities, do not create volumetrically significant weathering in the protolith. Above 38 m depth, biotite begins to oxidize, micro-cracks form around the biotite, and plagioclase, the most abundant weatherable mineral, begins to dissolve and create higher porosity. Above the same depth (38 m depth), seismic refraction velocities decrease and sonic velocities fluctuate markedly, consistent with the presence of fractures and increasing chemical weathering that opens up matrix porosity. Thus we surmise that weathering reactions are limited to the immediate vicinity surrounding fractures below 38 m but penetrate further into the surrounding rock at shallower depths.

Throughout the weathered and fractured bedrock layer, there is a clear upward increase in yellow hue in the rock, which we interpret as a gradual upward increase in chemical weathering (Fig. 1). We suggest that both time and reactive fluid transport are important here. In an eroding landscape, rock closer to the surface has spent more time in the zone where oxidizing fluids penetrate, increasing the extent of chemical weathering. Therefore, an upward increase in weathering extent is a natural consequence of interactions of regolith and reactive fluids during exhumation towards the surface. This progressive exposure effect is amplified by increasing contact with corrosive meteoric waters as fractures open and water percolates downward by microporous flow. The observed peaks in weathering intensity around visible fractures (Fig. 2) suggest weathering aided by advection, with water transported downward along fractures into the deep CZ, further promoting chemical weathering.

Data presented here from the Calhoun CZO significantly expand on similar geochemical features observed in other granitoid rocks^33^ by showing the tight linkage between chemical weathering and fracturing throughout the regolith of 38 m. Our results also corroborate many other studies of water-rock interaction in a variety of locales by showing that oxidizing fluids flow through the deep CZ in fractures in granitoid terrain. Discrepant high-frequency sonic velocities and low-frequency surface refraction velocities indicate that weathered bedrock contains fractures (at scales of 10’s of m) that reduce surface seismic velocities but have only localized effects (at individual fractures) on downhole sonic velocities. Borehole images reveal both planar fractures (of possibly tectonic origin) and non-planar fractures (possibly from chemical weathering). Vertical changes in geochemical and borehole image data reveal a downward decrease in weathering extent suggestive of the combined effects of downward-percolating reactive waters and additional time spent in the critical zone by rocks closer to the surface.

## Supplementary information

Supplementary Information

Supplementary Dataset 1

Supplementary Dataset 2

## Data Availability

The geochemical and geophysical datasets generated and/or analyzed during the current study are included in the Supplementary Information files in spreadsheet form. Raw geophysical logging data are available for download from https://www.hydroshare.org/resource/a95c034bc59a42efb25ff1e7ff888b22/. Surface seismic refraction data are available at https://datacorral.uwyo.edu/repository/M2RP4Q.
