# Supplementary material for: Links between physical and chemical weathering inferred from a 65-m-deep borehole through Earth’s critical zone
Source: Sci Rep. 2019 Mar 14;9:4495. doi: 10.1038/s41598-019-40819-9 (PMC6418305; doi:10.1038/s41598-019-40819-9)
Supplement: Supplementary file 1 — Supplementary Information [file 41598_2019_40819_MOESM1_ESM.docx]

**Supplementary Information**

**Links between physical and chemical weathering inferred from a 65-m-deep borehole through Earth’s critical zone**

W. Steven Holbrook^1,2^, Virginia Marcon^3^ , Allan R. Bacon^4^, Susan L. Brantley^3^, Bradley J. Carr^1^, Brady A. Flinchum^1^, Daniel D. Richter^5^, and Clifford S. Riebe^1^


^1^Dept. of Geology and Geophysics, University of Wyoming, Laramie, WY 82071
^2^Dept. of Geosciences, Virginia Tech, Blacksburg, VA 24061
^3^Earth and Environmental Systems Institute and Dept. of Geosciences, Pennsylvania State University, University Park, PA 16802

^4^Soil and Water Sciences Department, University of Florida, Gainesville, Florida 32611
^5^Nicholas School of the Environment, Duke University, Durham, NC 27708

**Contents: Supplementary Information**

List of Supplementary Tables ……………………………p. 3

Data Acquisition ……………………………………….p. 4

Drilling and sampling

Surface seismic refraction

Borehole logging

Rock Physics Modeling …………………………………p. 7

Geochemical Analyses and Calculations ……………….p. 8

Sample processing

Geochemical analyses

Mass balance calculations

Mineral model

Lithology Below 55 m ………………………………….p. 14

Fourier Analysis of Optical Borehole Images …………..p. 15

Alternative Calculations of Chemical Weathering ……...p. 19

Supplementary Figures …………………………………p. 21

**Supplementary Tables**

Additional supplementary data are included in table form in two separate files:

File “Geochemical_Data.xlsx” contains:

Supplementary Table S1. Bulk geochemical analyses (weight %).

Supplementary Table S2. Tau values assuming Zr is immobile.

Supplementary Table S3. Tau values assuming Ti is immobile.

Supplementary Table S4. Bulk geochemistry including >2 mm fraction (weight %).

Supplementary Table S5. Additional chemical analysis on <2 mm fraction (𝜇mol/kg) and corresponding 𝜏_j,i_ values.

Supplementary Table S6. Calculated mineral percentages and corresponding 𝜏 values.

Supplementary Table S7. Chlorite oxidation used for mineral model.

Supplementary Table S8. Alternative chemical weathering model: Tau values assuming Ti is immobile and that the sample at 32 m depth represents protolith.

File “Borehole_Geophysical_Data.xlsx” contains:

Supplementary Table S9: P-velocity from sonic log

Supplementary Table S10: Water content from borehole NMR log

Supplementary Table S11: Total gamma and spectral gamma log results

Supplementary Table S12: Calculated grayscale and yellow hue from optical borehole images

Supplementary Table S13: Fracture density calculated from acoustic borehole imager

Supplementary Table S14: Porosity calculated from sonic velocity log using rock physics model

Supplementary Table S15: Vp, Vs and density values used in rock physics model

**Data Acquisition**

*Drilling and sampling***.** The Calhoun borehole was drilled in 2010 in a pasture at coordinates 34.60690˚N, 81.72374˚W, approximately 3 km from Holcomb’s Branch tributaries that drain the main study area of the Calhoun CZO^17^. Three intact cores were collected with a Geoprobe to a depth of 6.1 m and composited by depth. Samples from below 6.1 m were collected as cuttings during drilling with air-rotary equipment using a three-wing auger and a roller cone bit. Cuttings were homogenized and subsampled in the field in 1.5 m intervals from 6.1 to 16.8 m, and in 3 m intervals from 30.5 to 67 m. Samples were not collected between 18 and 30 m depth. Above 18 m the borehole was cased to prevent collapse in the zone inferred by drillers to be “unconsolidated.” The water table at the time of drilling (June 2010) was at 5.5 m; during two downhole logging campaigns, the water table was at 4.4 m (April 2014) and 2.5 m (February 2016) depth. The average depth of the water table from 2014 to 2016 is 4.07 m.

*Surface seismic refraction***.** Refraction results from this site were presented in ref. 13 (their Fig. S7). We acquired two perpendicular, ~240-m-long seismic lines that intersected at the borehole location. Data were recorded on 96, 4.5-Hz vertical-component geophones spaced at 2.5 m intervals. Shots were fired at 10 m intervals along the line by striking a 20 x 20 x 2 cm stainless steel plate with a 5.4 kg sledgehammer. P-wave first arrival times were manually picked on all shots and inverted for P-wave velocity structure using a Matlab-based travel time tomography code^34^.

*Borehole logging***.** We logged the Calhoun borehole on two separate occasions; in April 2014 we obtained caliper, sonic velocity, natural gamma data and in February 2016 we obtained spectral gamma, optical borehole imager (OBI), acoustic borehole imager (ABI) and nuclear magnetic resonance (NMR) data. The NMR data were acquired with a Vista Clara, Inc., Javelin™ JP350; all other logs were acquired using the slimline tools of Mount Sopris Instruments.
 Borehole width carries information about the presence of fractures, highly weathered zones, and other irregularities. We measured borehole width, and thus created a caliper log, at 1.5 cm vertical intervals using a Mount Sopris Instruments QL40 three-arm caliper.
 Our sonic velocity log quantifies the P- and/or S-wave velocities using a high-frequency acoustic transmitter and one or more receivers. We used the Mount Sopris Instruments QL40FWS full waveform sonic tool, which has a 15 kHz source and three receivers on which full-waveform returns are recorded.
 We acquired both natural gamma and spectral gamma logs down the hole (Fig. S1). A natural gamma log measures the rate of naturally occurring, gamma-particle-producing radioactive decay (principally ^40^K, ^232^Th and ^238^U) in a formation and thus provides information about lithological and geochemical changes. Spectral gamma logs use the contrasting energy spectra to separate the contributions of ^40^K, ^232^Th and ^238^U. We used the Mount Sopris QL40-GR natural gamma probe (in April 2014) and the QL40-SGR spectral gamma probe (in February 2016). The measurement units for the natural gamma probe are in API (American Petroleum Institute) units, which provide a relative measure of gamma decay in the formation. Units for the spectral gamma probe are weight percent for ^40^K and ppm for ^232^Th and ^238^U.
 We used the Mount Sopris QL40-OBI-2G optical televiewer, which captures high-resolution (up to 1800 pixel) photographs of the borehole wall, and creates an optical borehole imager (OBI) log. Our images were recorded at 990 pixels so that data recording rates could keep pace with the continuously moving tool. Images are unwrapped, 360˚ views of the cylindrical borehole wall. The tool has a built-in, three-axis magnetometer and a three-axis accelerometer to provide orientation referenced to true north throughout the hole. Images are acquired with a fisheye lens and digital image sensor. The light source comes from LED’s with an intensity of 750 lm and a color temperature of 5600 K.
 We conducted color analysis on the optical borehole images by reading RGB (red, green, blue) values from jpeg images using Matlab’s Image Processing toolbox. We calculated an average gray scale at each depth (approximately 0.9 mm vertical interval) by taking the mean RGB value, (R+G+B)/3. We calculated a “yellow/brown” hue at each depth by (R+G)/2 - B. We use the yellow hue as a qualitative indicator of the degree of rock weathering, since the protolith is largely gray in color and yellow/brown colors indicate staining due to chemical weathering (Fig. 1).
 Acoustic borehole imagers (ABI) send acoustic pulses out to the borehole wall and record the amplitude and travel-time of the return. This provides detailed information on the shape and hardness of the borehole wall, information that is often interpreted in terms of fractures. Fractured rock tends to have longer travel times (expanded wall radius) and weaker amplitudes (softer rock). We acquired ABI data with the Mount Sopris Instruments QL40ABI. We use ABI travel times and amplitudes to infer fracture density. A user-selected threshold for anomalously long travel times and weak amplitudes is used to assign a binary value to each pixel of the borehole wall (1=fracture, 0=intact rock). Inferred fractures from this approach were checked visually against the OBI data for consistency in interpreted fractures. At each level of the image, the total percentage of “1” values was compiled as a measure of fracture density. Due to noise in the data, this method likely overestimates the true fracture densities, but the relative changes up and down the hole are informative (e.g., see Fig. S2).

Nuclear magnetic resonance (NMR) data provide direct information about water content and pore space properties by exciting hydrogen atoms in water with a tuned, alternating magnetic field and measuring EM signals emitted by their precession back into alignment with a static magnetic field. For a full review on NMR methods the reader is referred to ref. ^35^, and for recent examples of the application to granite-cored critical zones, see refs ^36,37^. Downhole NMR data were acquired with a Vista Clara Javelin JP350 instrument which measured water in four cylindrical shells extending from the center of the borehole. The instrument uses a Carr–Purcell–Meiboom–Gill (CPMG) pulse sequence^38,39^ to obtain a measurement every 0.5 m. Data were processed with Vista Clara – Javelin Processor 3.41 software and inverted with Vista Clara – Javelin Interpretation 1.5 to produce estimates of water content and pore size distribution. Here we present only the water content estimates, which are equivalent to porosity in fully saturated pores, as expected below the water table.

**Rock physics modeling**

P-wave velocities can help constrain porosity in rocks, provided that the bulk mineral composition is reasonably well known and that an appropriate rock physics model is applied. Holbrook et al. (ref. ^40^) used a contact theory model to estimate porosity in saprolite in the CZ and compared those estimates to measured porosity in push-core samples. We estimated porosity in the weathered bedrock and protolith from sonic velocity using a differential effective medium (DEM) model that simulates the effects of cracks on a solid medium. DEM theory assumes that the elastic properties of a material can be calculated by integrating infinitesimal changes in an elastic solid^41-45^. We used the model described by ref. ^43^, which assumes penny-shaped cracks in crystalline rocks. For the host material, we assume a granite with a bulk modulus of 56 GPa, shear modulus of 31 GPa, and a density of 2.64 g/cm^3^ (Vp ~ 6000 m/s at zero porosity). We used porosity and velocity measurements made on granites from literature^46-48^ (Fig. S3a) to calibrate the DEM model. The aspect ratio of the crack is the primary calibrating parameter in the model since it cannot be directly measured ^43^. The aspect ratio that provided the best fit to the literature data was 0.016 (Fig. S3a). To validate the model we plot the predicted porosities against measured porosities and found that they were correlated (R2 = 0.84) with a slope close to unity (Fig. S3b). We simulated the effects of air-, clay- or water-filled cracks to create three plausible relationships between porosity and velocity (Fig. S3a), which we can use to validate NMR measurements and link the geophysical data to the geochemical data.

**Geochemical Analyses and Calculations**
*Sample processing.* Samples shallower than 6.1 m were sieved to remove the >2 mm fraction following standard soil sampling protocols. Mineral material >2 mm diameter was found in only eight of the 45 sieved samples. Where present, the >2 mm mineral material averaged 7% and ranged from 1-11% by volume. For all depths deeper than 6.1 m, bulk samples were collected and analyzed without sieving. Bulk chemistry was estimated from both raw data and by assuming that the missing >2 mm fraction (where present) was entirely SiO_2_. This correction of the upper 0-6.1 m samples minimally affects the results (Table S4).

Thin sections were made from cuttings sampled at 32, 35.1, 41.2, 56.4, and 59.5 m (depths reported as mid-point of sampling interval). Additional epoxy mounts were made for ground sample (<150 μm) at 7.6, 12.9, and 18.3 m depths and observed in optical and back-scattered electron microscopes.

*Geochemical analyses.* Samples were digested and analyzed for elemental concentrations by inductively coupled plasma (ICP) atomic emission spectroscopy (AES) and atomic absorption spectroscopy (AAS). Aliqouts were analyzed for U and Th concentrations by ICP mass spectroscopy (Table S5). Ferrous iron was analyzed using a titration method described by ref. ^49^. A correction was applied to account for oxidation of U and Mn assuming U oxidized from 4+ to 6+ and Mn from 2+ to 3+ redox state [Fe(II) – (Mn) – (U/2)].

Inorganic carbon was determined in the Biogeochemistry Laboratory, Department of Crop and Soil Sciences, Penn State using a LiCOR CO_2_-H_2_O analyzer (LI-7000) following methods reported by ref. ^27^ and adapted from ref. ^50^. Approximately 1 g of rock (ground to 100-mesh) was placed in 120 ml serum bottles, sealed, and acidified with 5 ml of 1N HCl. The samples were shaken for 24 hours to ensure complete dissolution of carbonates within the rock sample. CO_2_ released from reaction with HCl was captured in the bottled headspace, and 1 ml of the headspace was sampled and analyzed. Three serum bottles were filled with only HCl and the headspace sampled to determine the background CO_2_ value. A standard curve was made by injecting known amounts of CO_2_ from gas tanks with either 970 of 10,300 ml/l of CO_2_. Each measurement was taken 3 times and averaged. Checks were run every at the beginning, middle, and end of the run. Accuracy was evaluated by analyzing known USGS standards (SGR-1 and G2) in the same manner as the samples. Errors were calculated by analyzing the standard 3 times and duplicating 2 samples. The spread in standards ranged from 8 to 17%, and sample duplicates had spreads of 12 to 15%.  Results of the calcite determinations are plotted in Fig. S4 along with grayscale and yellow hue from the OBI data.

*Mass balance calculations.* We estimated chemical depletion of samples of drill cuttings by comparing the changes in element concentrations relative to protolith using mass transfer coefficients inferred from mobile and immobile element concentrations (e.g., ref. ^51^). Here we use Ti and Zr as immobile reference elements, under the assumption that they are both only present in largely insoluble minerals in granitoid rocks. The composition and variability of protolith is difficult to define in chemical depletion studies. Here, we defined protolith by averaging the five chemically analyzed samples retrieved between 38 and 53 m (Tables S2 and S3). We used Equation 1 to calculate *τ_i,j_*, the mass transfer coefficient, which is the fractional loss or gain of an element (subscript *j*) with respect to the immobile element (subscript *i*) in the protolith^51,52^.

$$\tau_{i,j}= \frac{C_{j,w}C_{i,p}}{{C_{j,p}C}_{i,w}}-1$$

Equation 1

Here *C* is concentration, and subscripts *p* and *w* denote the protolith and weathered material, respectively. If an element is neither lost nor gained with respect to the immobile element then *τ_i,j_* = 0. Negative and positive *τ_i,j_* values imply loss and gain, respectively. A value of chemical depletion for the entire sample or “bulk tau” can also be calculated as shown in Equation 2.

$$\tau_{bulk}= \frac{C_{i,p}}{C_{i,w}}-1$$

Equation 2

The absolute value of *τ_bulk_* should be equal to the total porosity *ϕ*, if (1) the rock weathers isovolumetrically, (2) the protolith has been accurately identified and has very low initial porosity, and (3) element *i* is completely immobile and has not been added during weathering. Under these conditions, bulk tau can be thought of as an estimate of the “chemical porosity” of the weathered rock.

*Mineral model.* A mineral model was developed in order to quantitatively determine mineralogy change with depth. The model was constrained by petrographic and SEM observations; XRD analyses; and EDS and microprobe mineral chemistry. Bulk elemental chemistry (in mol/kg; Table S4) was allocated to each mineral based on mass balance. The elements are distributed to each mineral as enumerated below.

All P was allocated to apatite, the only P-bearing mineral in the system, and Ca was partitioned based on stoichiometry. This consumes some Ca from the bulk reservoir.

Albite is the only Na-bearing mineral; therefore, all Na was distributed to albite. The Na:Ca ratio determined by microprobe analyses was used to calculate a general albite formula, (Na_0.87_Ca_0.13_)Al_1.13_Si_2.87_O_8_, and Ca, Al, and Si was partitioned stoichiometrically relative to Na.

Calcite was measured directly consuming some additional Ca.

The remaining Ca was allocated to epidote using a general formula of Ca_2_Al_3_(SiO_4_)_3_(OH) and Al and Si were distributed accordingly.

Pyrite is assumed to be the only S-bearing mineral. Sulfur was measured directly and Fe allocated based on stoichiometry.

All of the Mg was allocated to chlorite and biotite based on an assumed ratio of 1:4. The ratio was determined by XRD and petrographic observations. The Mg:Fe ratio in biotite and chlorite was determined by microprobe and used to calculate mineral formulas for the parent material. A general biotite formula of K(Mg_1.05_Fe_1.95_)AlSi_3_O_10_(OH)_2_ was used in the mineral model, and K, Fe, Al, and Si were allocated to biotite stoichiometrically relative to Mg. A general chlorite formula of Mg_1.5_Fe_3.5_Al_2_Si_3_O_10_(OH)_8_ was used and Fe, Al, and Si were allocated based stoichiometrically relative to Mg assuming all ferrous iron in the parent.

Fe(II) was evaluated by titration and Fe(III) calculated by difference. 10% Fe(III) was attributed to ilmenite, which consumes some Ti.

Some Al was distributed to muscovite based on the observed muscovite and plagioclase ratio (0.03:1) determined by XRD. A general formula of KAl_3_Si_3_O_10_(OH)_2_ was used to allocated K and Si, while the remaining K was allocated to orthoclase and Al and Si were distributed accordingly.

Al was allocated to pyrophyllite based on Pyrophyllite/plag ratio from XRD analyses.

All Zr was allocated to zircon consuming some Si; the remaining Si was allocated to quartz; the remaining Ti was allocated to rutile; and Mn was allocated to Mn-oxide.

The remaining Fe(III) was assigned to magnetite and the proportional mole ratio of Fe(II) was allotted, and the remaining Fe was attributed to hematite.

Results of the mineral model analysis are summarized in Table S6. The validity of the model was checked based on the relative quartz content observed in microprobe elemental maps and quantitative XRD results.
 As the rock begins to weather between 35 and 38 m, the model was adjusted accordingly. XRD data shows that pyrophyllite and muscovite were consumed above 27 m and therefore, they were removed from the model. When plagioclase dissolution begins (35 m), secondary kaolinite is added to the model to account for excess Al and Si. Oxidation reactions occur at 35 m so additional minerals, altered biotite and chlorite, were added to the model to account for Fe(II) oxidizing to Fe(III). The amount of Fe oxidation in biotite was evaluated by the change in K: As Fe(II) is oxidized to Fe(III), K is lost from biotite to maintain charge-neutrality. Oxidation of biotite is assumed to occur over a small interval from 38 to 35 m, and all the biotite in the sample was oxidized equivalently. 30% of K was lost between 38 and 32 m, which was used to calculate a general altered biotite formula:
 K_0.68_(Mg_0.9_Fe(II)_1.78_Fe(III)_0.32_)AlSi_3_O_10_(OH)_2_

The amount of altered chlorite was determined by the loss in Mg, which ranges from 0.17 to 0.30 over the 38-9 m depth interval and thus constrains the amount of oxidation. This calculation assumes that biotite alteration did not result in the loss of Mg, consistent with observations from microprobe analyses. The formulas used for oxidized-chlorite are provided in Table S7.

In the upper 18 meters, where weathering is the greatest, additional clays were added to the model based on XRD results. Illite (K_0.8_Al_2.1_Fe^3+^_0.9_Si_3_O_10_(OH)_2_) was added as a by-product of plagioclase and muscovite weathering. The illite:quartz ratio determined by XRD was used to allocated the elements proportionally. Vermiculite was added to the model to account for excess Mg. Above 13 m, montmorillonite (Na_0.9_(Al_1.1_Mg_0.9_)Si_4_O_10_(OH)_2_) was added to account for excess Na in the system based on Ca available to attribute to plagioclase.

**Lithology below 55 m**

As noted in the caption to Figure 1, we defined the protolith to be a zone of relatively homogeneous rock chemistry and physical properties from about 41-53 m. (Sample depths are averages of 3-m-thick zones and plotted at their midpoint depths.) Below about 55 m, optical and physical properties change drastically, suggesting a different lithology. As seen in the optical borehole image (Figs. 1 and S5), the rock below 55 m contains abundant layers of light-colored rock, which often have high values of yellow hue, suggesting the yellowish coloring may be intrinsic and not an indication of weathering in this rock – an inference supported by the observations that yellow hue no longer has an inverse correlation to sonic velocity, and that NMR-measured porosities here are the lowest in the hole. One prominent such layer is 1.5 m thick, extending from 59.5 to 61 m depth. Outside the light-colored layers, the rock is on average much darker than it is above 53 m (Fig. 1), and below 61.5 m the rock has a “vuggy” appearance with a very rough borehole wall. Although major-element tau values remain near zero for the most part, there are large deviations in biotite and Fe(II) (Fig. 1), as well as in calcite (Fig. S4). For all these reasons, we interpret the rock below about 55 m as a different lithology underlying the gray-colored, relatively homogeneous granitoid above 55 m.

**Fourier Analysis of Optical Borehole Images**

The upward increase in yellow hue in the weathered bedrock layer is a potentially significant observation in the optical borehole images at our site. However, the images in the upper few meters (18-24 m) of the layer appear slightly blurry, suggesting turbidity in the water in the borehole. If the blurriness is caused by turbidity, and if the particles causing the turbidity have a yellow/brown color, the observed upward increase in yellow hue might merely reflect an upward decrease in water clarity, rather than a property of the wall rock. To assess this possibility, we conducted Fourier domain (horizontal wavenumber) analysis of the images. We hypothesized that if the upward increase in yellow hue is due to turbidity, then we should see a similar gradual upward decrease in high-wavenumber power in wavenumber spectra of the optical borehole images. Horizontal wavenumber power spectra contain information about the clarity of images and also the grain size and uniformity of the rock exposed in the borehole wall, as we will show below.
 To conduct the analysis, we calculated the power spectral density in the horizontal wavenumber domain of optical images of the borehole wall using the “periodogram” function in Matlab. We first converted the color jpeg images to grayscale images by calculating (R+G+B)/3, where R, G and B are the red, green and blue components of the color jpeg image, respectively (Fig. S6). Next, we extracted grayscale values at each depth, normalized so that 0=black and 1=white (Fig. S7). We then calculated power spectral density in the wavenumber domain at each depth, and summed the spectra over 15 cm depth intervals to improve signal-to-noise before plotting (Fig. S8).
 Fig. S8 shows that there are distinct changes in high-wavenumber spectral energy of the optical images with depth, with (for example) low energy at 19-22 m and higher energy at 32 m. Low energy at high wavenumbers, as observed at 19-22 m, could be due to (1) blurry images, which reduce the color contrasts between grains, (2) changes in grain size in the wall rock, or (3) lithological changes that comprise more uniform grain colors.
 To demonstrate the effect of a blurry image, we artificially blurred the optical borehole image at 31 m depth by applying a median filter. The spectrum of the blurred image shows a distinct decrease in energy at most wavenumbers, especially at higher wavenumbers (Fig. S9). This demonstrates that a blurry image – for example, due to turbid water in the borehole – will have lower spectral energy than a clear image of the same rock.
 To estimate the depth at which image clarity changes in the OBI data, we calculated the mean spectral level in each 15-cm depth window from 1 to 5 cycles per cm. This wavenumber range would easily capture any blurring in the images (Fig. S9) but avoids the noise floor at the highest wavenumbers (Fig. S8). We defined “image smoothness” as –log_10_(E), where E is the mean spectral density of the wavenumber spectra from 1 to 5 cycles per cm. The resulting estimate of image smoothness shows striking changes with depth (Fig. S10).
 At first glance it might appear that the upward increase in yellow hue from 33 m to 18 m is matched by a similar increase in image smoothness, which in turn might support the idea of an upward decrease in image clarity as the cause of the yellow color. However, upon close inspection it is obvious that the high-wavenumber spectral energy is not primarily reflecting image clarity, for several reasons: (1) The OBI images show no visible evidence of turbidity below about 23 m (main text, Fig. 1), yet the image smoothness continues to change all the way down to 33 m. Thus, while the overall high values of smoothness from 18 to 22 m may in part reflect turbidity, the upward increase in smoothness from 33 to 22 m cannot. (2) A strong change in smoothness occurs at 32 m depth, even though the images above and below that depth are not blurry. Rather, the change at 32 m is associated with a lithological change, as it occurs at the same depth as a change in color. A similar change at 43 m depth also reflects a lithological change (discussed further below). (3) When examined in detail over short depth intervals, yellow hue and image smoothness do not co-vary as predicted by the turbidity hypothesis. For example, from 30 to 32 m, yellow hue increases gradually upward, but image smoothness is constant; from 21 to 22.5 m, yellow hue increases upward but image smoothness decreases. Thus, image smoothness must be primarily indicative of something other than turbidity in the well water.
 Instead, the image smoothness appears to reflect three properties of the wall rock: Grain size, mineralogical variability, and chemical weathering itself. Grain size would be expected to influence wavenumber spectra, since color changes occur at a spacing determined by grain size: larger grain sizes would produce less energy at high wavenumbers. Evidence for this effect is shown in Fig. S11: the rock at 25 m depth has visibly larger grain sizes than the rock at 33 m. As expected, the spectra at 33 m show greater energy at high wavenumbers (lower “smoothness”; Fig. S9). Mineralogical variability would also be expected to influence wavenumber spectra, since the “energy” in the images reflects lateral changes in grain color (e.g., Fig. S7): a more uniform rock will have lower spectral energy. Evidence of this effect can be seen in Fig. S12, by comparing the images at 31 and 41 m depth. The rock at 41 m is more uniform (and darker, as discussed in the main text) than at 31 m; the corresponding spectra show lower energy (greater smoothness) at 41 m (Fig. S10). The smoothness measure is thus a sensitive indicator of lithological boundaries in the rock, highlighting both the previously identified lithology change at 34 m depth, as well as another boundary just above 44 m between relatively uniform dark rock and an underlying unit that has numerous light-colored intrusions (main text, Fig. 1). Finally, it is possible that the yellow hue and the high-wavenumber energy are both changed by weathering processes: chemical weathering would be expected to attack mafic (darker) minerals first, which might decrease the grayscale variability of the image, thus leading to lower energy in wavenumber spectra. Evidence for this is seen in the association of peaks in yellow hue with increases in image smoothness in highly weathered zones (e.g., Fig. S10 at 20.3, 22.3, 28.5 and 32.5 m).
 In summary, careful analysis of the optical borehole images shows no evidence that the upward increase in yellow hue is due to turbidity in the water filling the borehole. Although we cannot completely rule out some potential influence of turbidity on the image color at the very shallowest levels (18-22 m), the overall upward increase in yellow hue appears to be an intrinsic property of the wall rock, not an artifact of the fluid in the borehole.

**Alternative Calculations of Chemical Weathering**

Estimates of elemental mass loss (*τ_i,j_*) depend critically on an assumed protolith or parent rock. The assumption of a single parent rock is especially challenging for a deep weathering profile, such as we present here. The calculations of chemical weathering presented in Figure 1 rely on the simple assumption of a single protolith with the composition of the relatively unweathered granite at depths of 40-53 m. However, as described in the text, the presence of an apparent boundary at 34 m depth, with slightly lighter-colored, more radiogenic rock above slightly darker, less radiogenic rock (Fig. S13), raises the possibility that the rock above this depth has a different protolith than that below. Although we lack sufficient data to fully resolve this question, it is worthwhile exploring what effect a different parent rock composition above 34 m could have on the calculated *τ_i,j_* values. We approach this by calculating an alternative model, assuming that the rock sample from 32 m depth, just above the possible boundary, represents unweathered protolith for the overlying section. The resulting tau values are included as Table S8. This calculation likely represents an extreme end-member model, since it by definition assumes no weathering at 32 m, even though the mineral model (Table S6) shows some weathering products at that depth (altered biotite and altered chlorite).

The *τ_i,j_* values for the alternative model (Model B), assuming the sample at 32 m represents the protolith composition, are shown in Figures S14B and S15B. Similar to the preferred model (Model A; Fig. S14A and S15A), elements dominantly present in plagioclase (Na and Ca) show major depletion (at least 50%) by 18 m depth (though the depletions are even greater for model A). There is also a rapid loss in K and Mg above 4 m depth in both models, likely related to the loss of orthoclase, biotite and clays (though again Model A shows more depletion than Model B). The major differences are that Model B predicts less chemical weathering overall in the saprolite for all elements (e.g., tau for K at 9.9 m depth is -0.08 in Model B but -0.5 in Model A). Moreover, at some depths Model B predicts mass gain of Si (tau ~0.1 at 1 m depth) and Al (tau 0.2-1.4 at depths from 2-6 m). What remains unclear in either model are the details of chemical depletion in most of the fractured bedrock layer, due to the lack of samples from that depth range.

**Supplementary Figures**


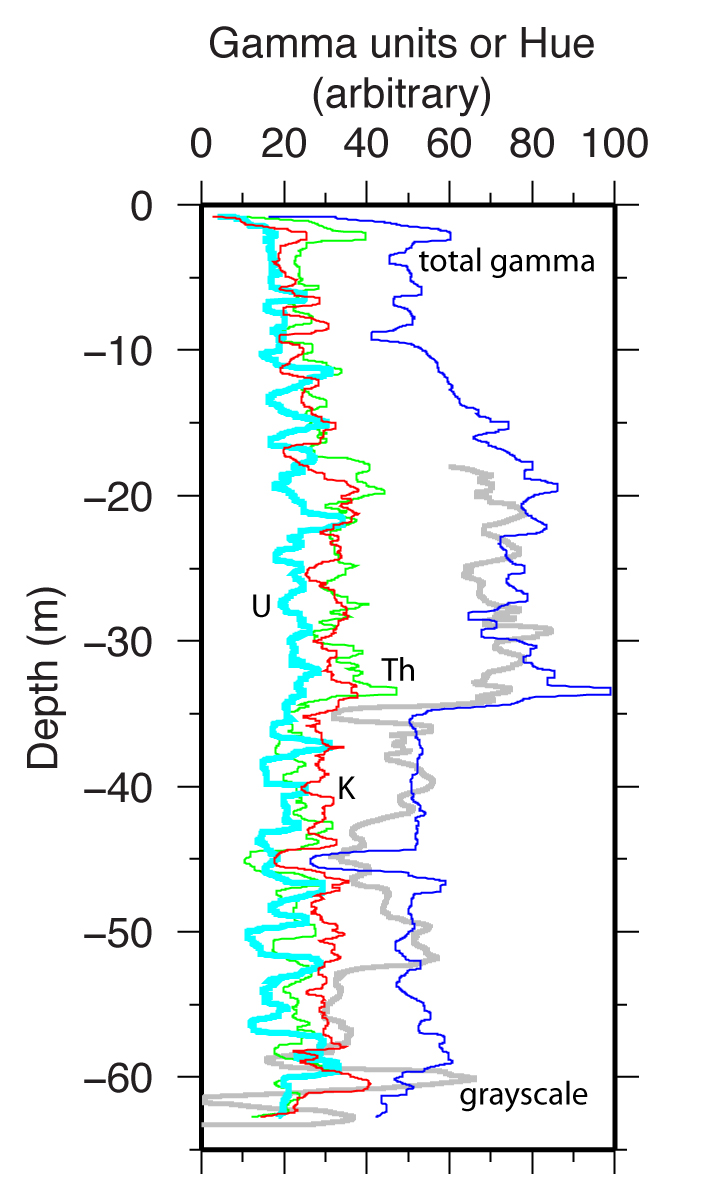


**Supplementary Figure S1 | Image and gamma log data.** Grayscale from optical image (gray), compared to total gamma log (blue) and spectral gamma logs for ^238^U (cyan), ^232^Th (green), and ^40^K (red). A clear downward change from lighter to darker rock coincides with a downward decrease in gamma activity. The spectral logs show that the decrease in gamma activity is controlled by decreases in ^232^Th and secondarily in ^40^K.


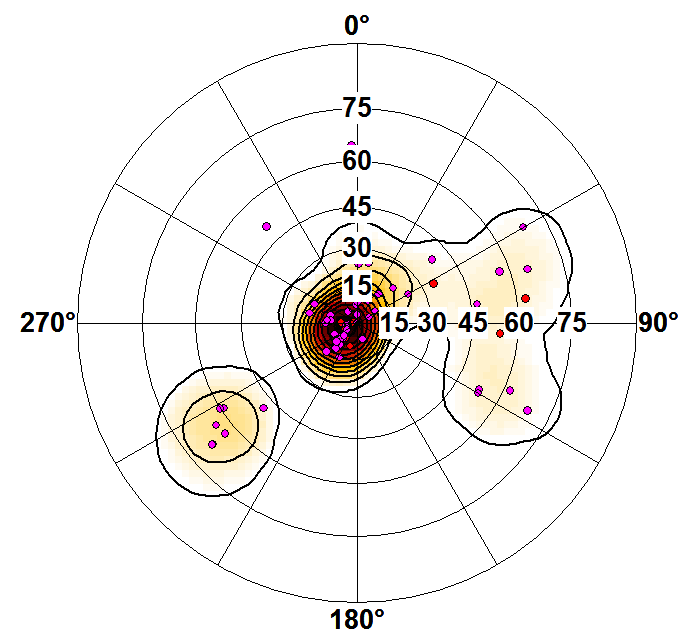


**Supplementary Figure S2 | Southern hemisphere Wulff plot of the Calhoun borehole.** Plot show fracture strikes and dips determined from the acoustic and optical televiewer data. Dots represent the projected poles of the fracture plane contoured by a Kamb method with a significant sigma = 2.  Most fractures (~ 77%) strike ~ N 30°E and are nearly horizontal (<15°) with a dip direction ~ N 60° W.


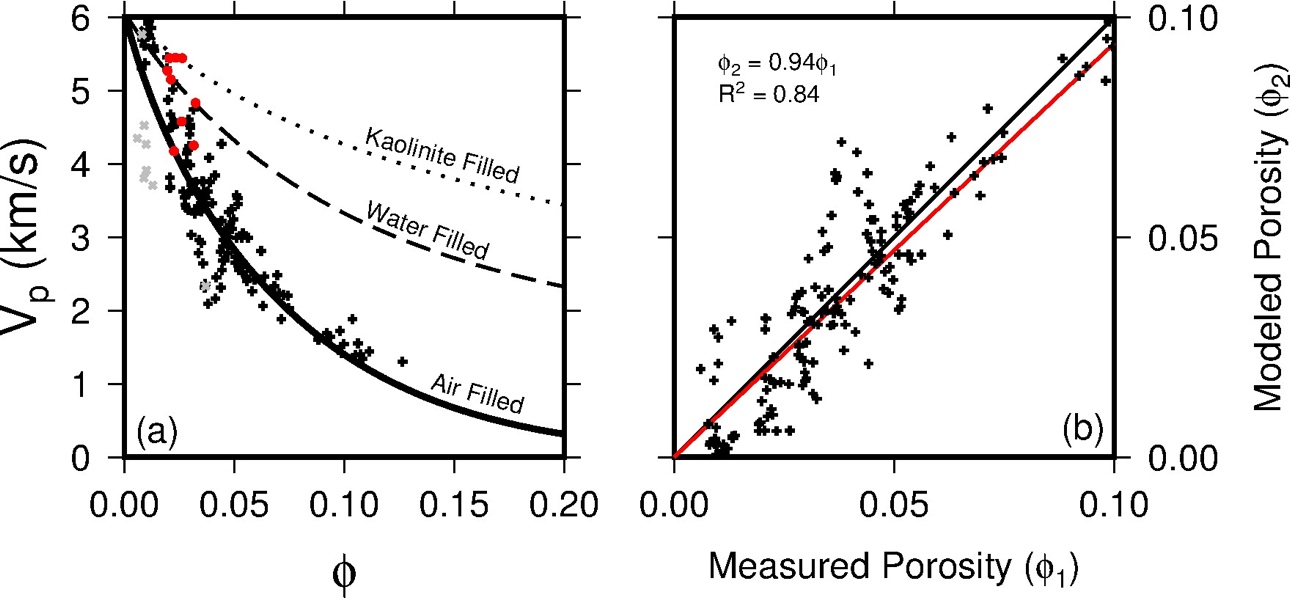


**Supplementary Figure S3 | Rock physics relationships.** **(a)** The relationship between porosity and P-wave velocity calculated using DEM model ^36^. The symbols represent measured porosity and velocity values from granites compiled from literature: black crosses are from ref. 39, gray x’s are from ref. 40, and red circles are from ref. 41. The thick black line shows the best fitting model (aspect ratio = 0.016) which was determined by minimizing the RMS between the measured and modeled data. The dashed line shows how the velocity porosity relationship changes if the fractures are filled with water (K = 2.25 GPa, G = 0 GPa) and the dotted line assumes Kaolinite (K = 1.5 GPa, G = 1.4 GPa) ^46^. **(b)** Cross plot showing the porosities predicted from the DEM model and the measured porosities. The black line is the 1:1 line and the red line is the least squares linear regression fit to the data, which had a slope close to one.


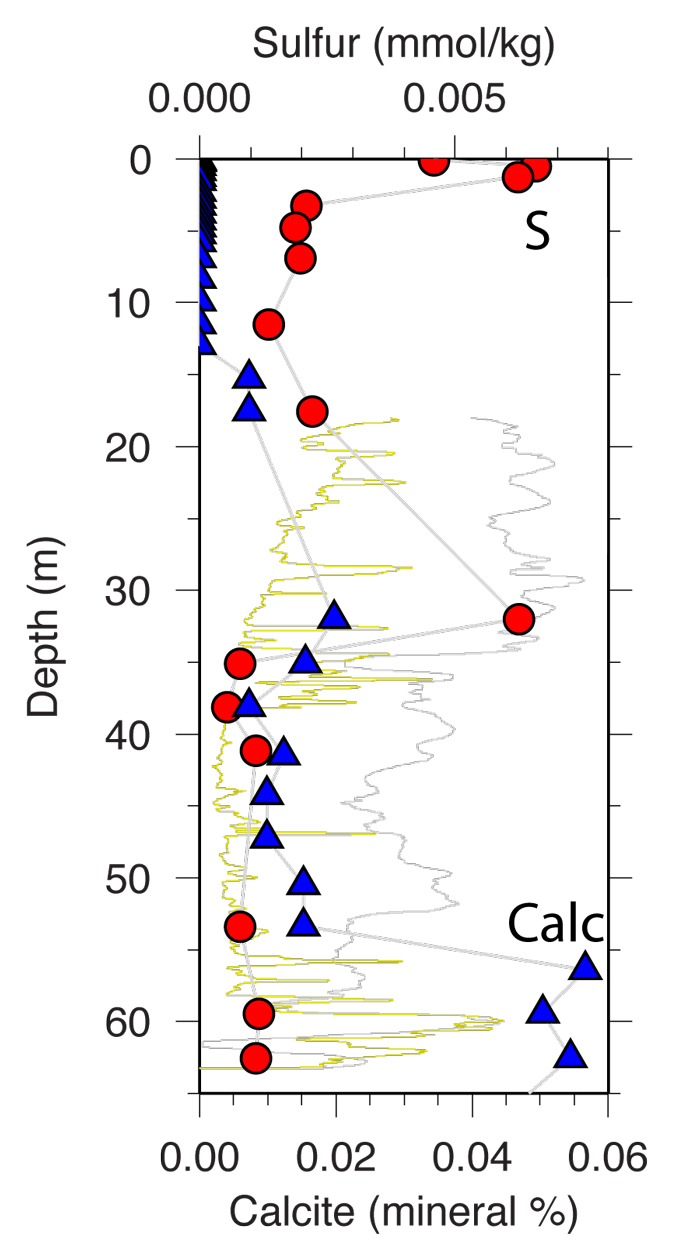


**Supplementary Figure S4 | Weathering from images and geochemical data.** Sulfur (S, red circles) and calcite (Calc, blue triangles) concentrations in comparison to yellow hue (brown line) and gray scale (gray line) from optical borehole images (arbitrary scale, see Fig. S1).


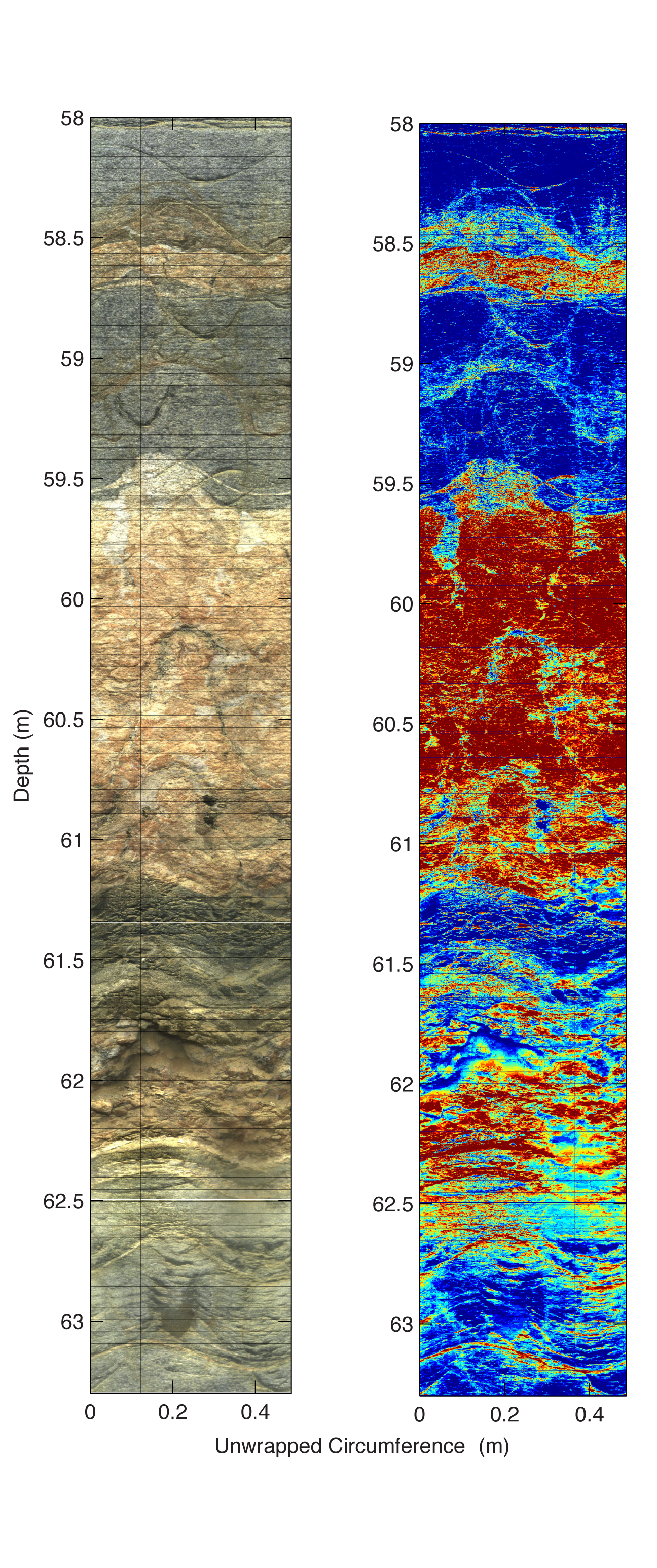


**Supplementary Figure S5 | Borehole images below 58 m.** Optical borehole image (left) and calculated yellow hue (right) of borehole wall below 58 m depth, plotted as in Figure 1.


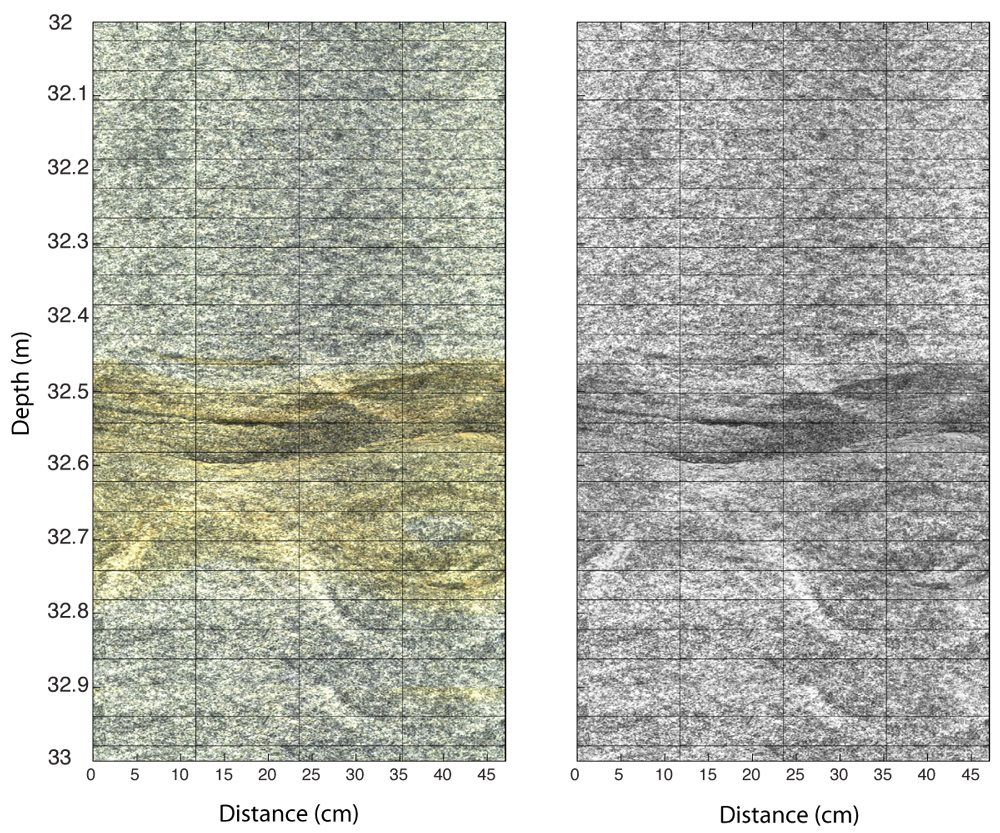


**Supplementary Figure S6 | Optical borehole image from 32 to 33 m depth.** Full RGB color **(*Left*)** and grayscale **(*Right*)**.


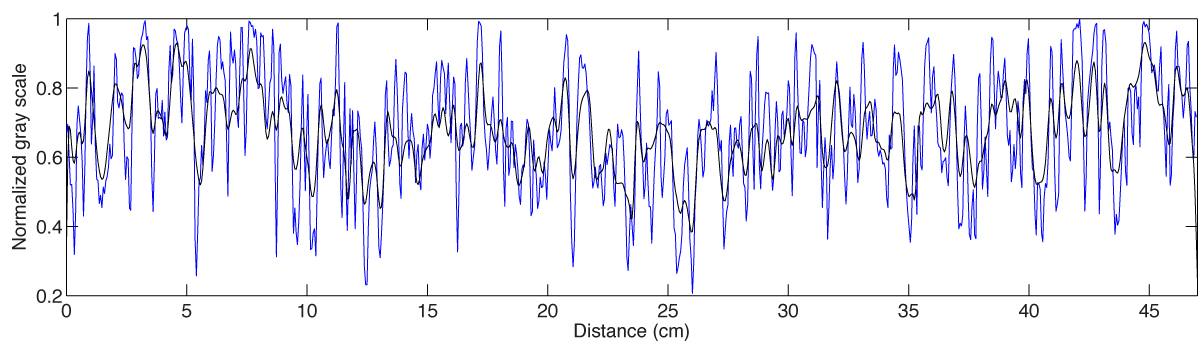


**Supplementary Figure S7 | Normalized grayscale from OBI at 31 m depth.** Grayscale ranges from black (0) to white (1). Trace shows data before (blue) and after (black) “blurring” by application of a median filter.


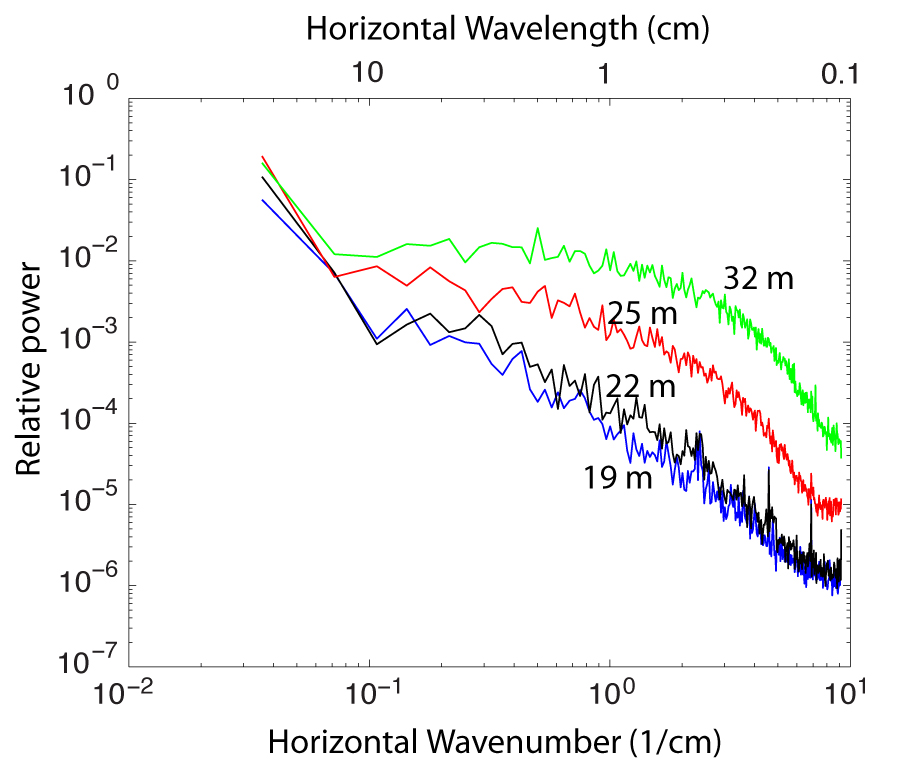


**Supplementary Figure S8 | Power spectral density of grayscale.** Different colors correspond to different depths.


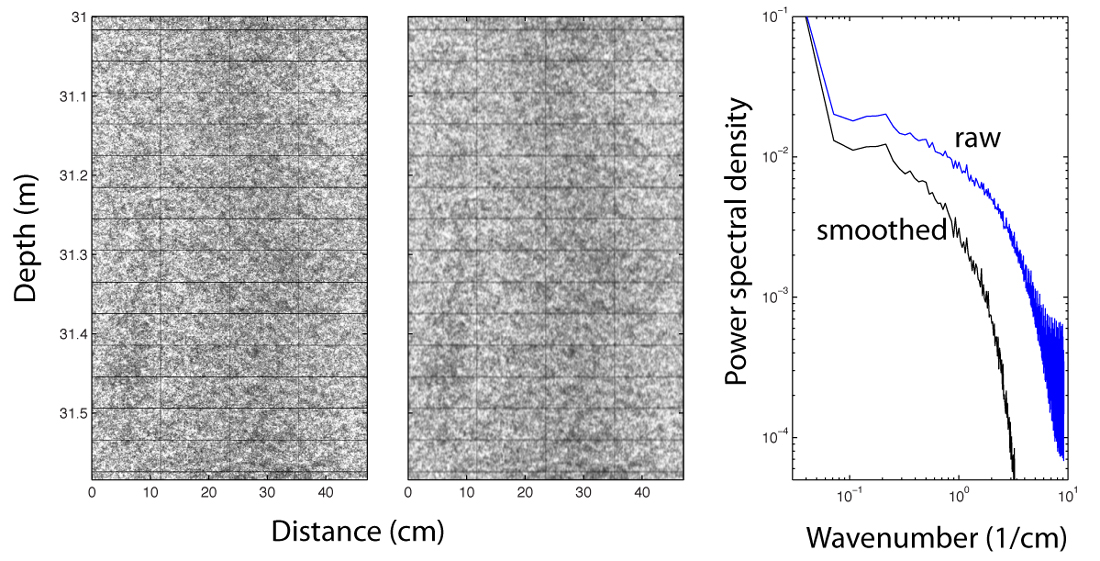


**Supplementary Figure S9 | Grayscale image analysis.** Grayscale of optical borehole image at 31-31.5 m **(*Left*)**, same image “blurred” by three consecutive applications of a spatial median filter ***(Center)***, and power spectral density of the image before (blue) and after (black) blurring **(*Right*)**.


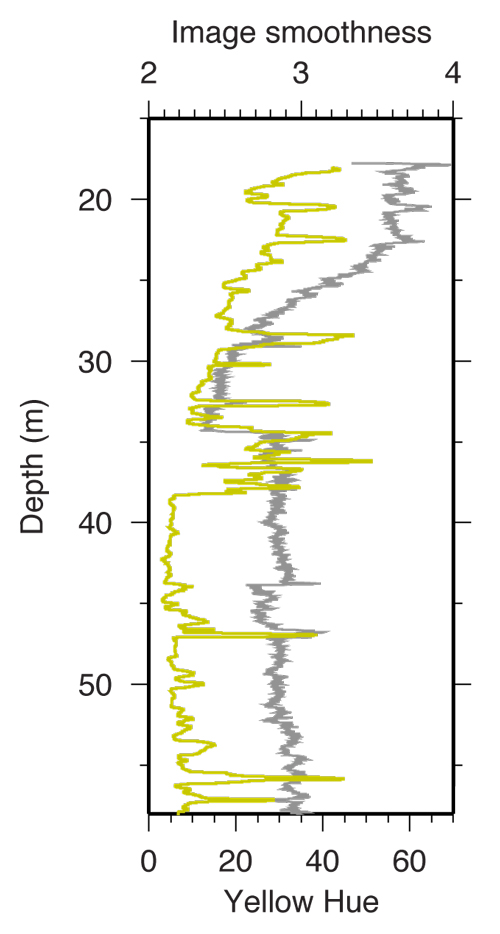


**Supplementary Figure S10 | Plot of yellow hue and image “smoothness” in optical borehole images.** Yellow hue (yellow line) is calculated as described in the main text and shown in Figs. 2, 3 and 4. Image smoothness (gray line) is the negative of the base-10 log of the mean spectral density at 1-5 cycles per cm in a 15-cm band centered at each depth point, calculated as shown in the examples above.


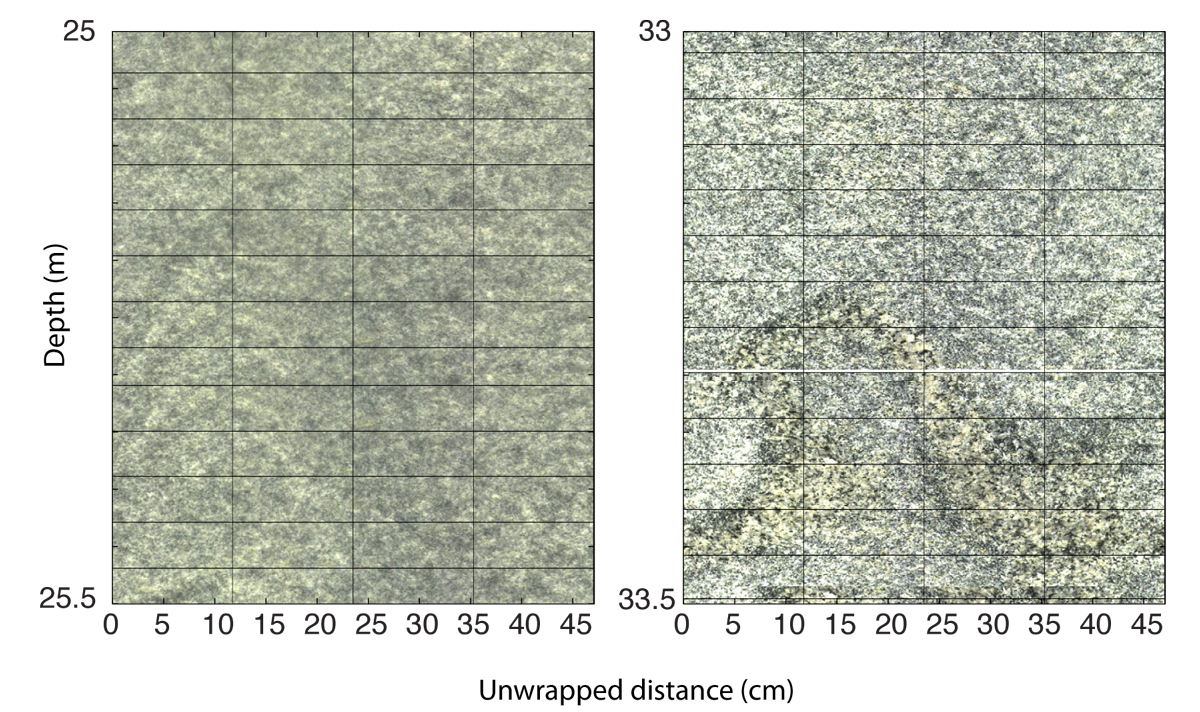


**Supplementary Figure S11 | Color images at 25 and 33 m depth, showing contrast in grain sizes.** The mean grain size of the rock decreases downward.


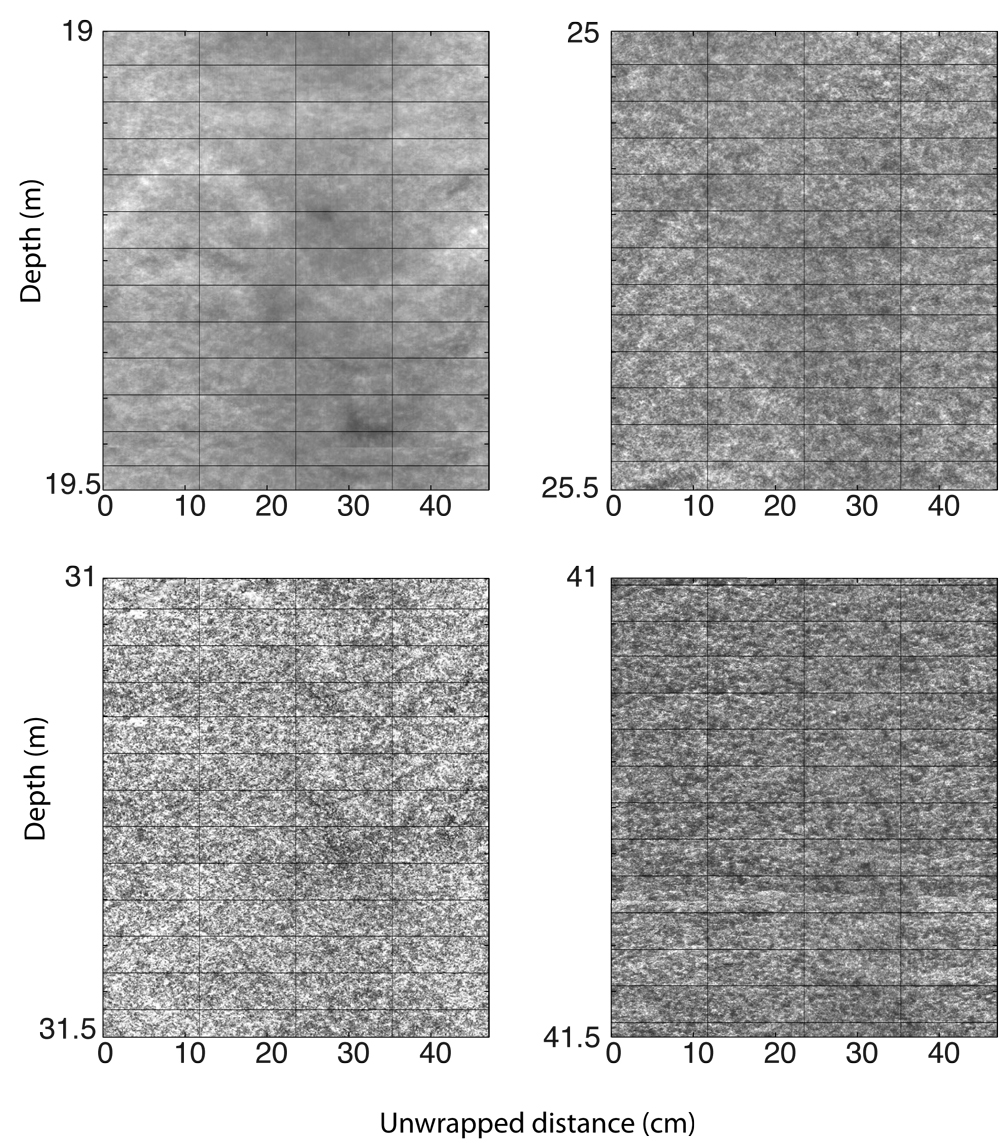


**Supplementary Figure S12 | Grayscale images at 19, 25, 31 and 41 m depth, showing contrast in grain sizes.** The mean grain size of the rock decreases downward.

**
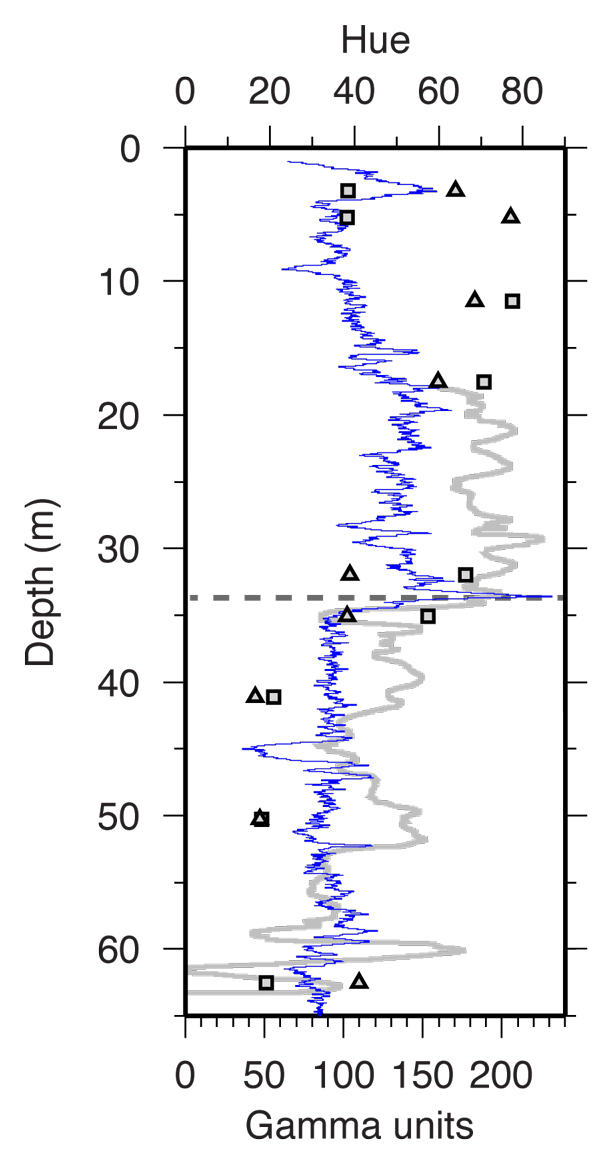
**

**Supplementary Figure S13 | Evidence for possible lithological boundary at 34 m depth.** Plot of natural gamma log (blue line, 1-m-width median filtered), grayscale color (gray line), and concentrations of U (triangles) and Th (squares). Dashed line shows location of a possible lithological boundary at 34 m depth, separating slightly lighter-colored, more radiogenic material above 34 m from slightly darker, less radiogenic material below 34 m.

**
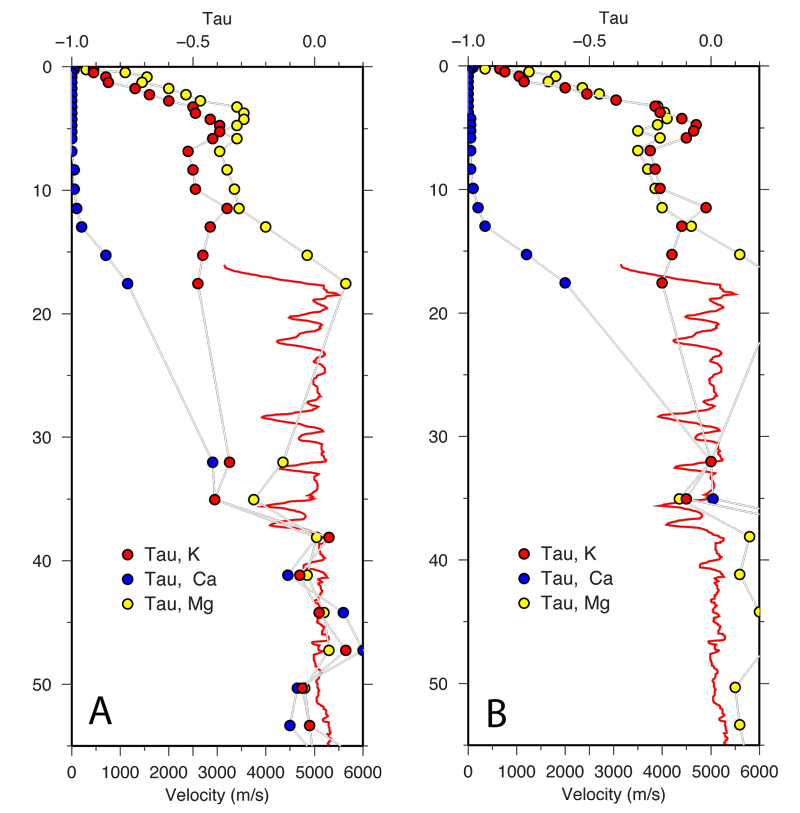
**

**Supplementary Figure S14 | Alternative models of chemical weathering.** Calculated chemical mass loss (tau) values for K, Ca, and Mg, assuming protolith is (A) the average composition of samples from 40-53 m depth, or (B) the composition of the sample at 32 m depth. The two models likely bracket the maximum variability expected for the calculated mass losses at this site. Sonic velocity is plotted to show extent of fractured bedrock layer (where sonic velocity markedly fluctuates).

**
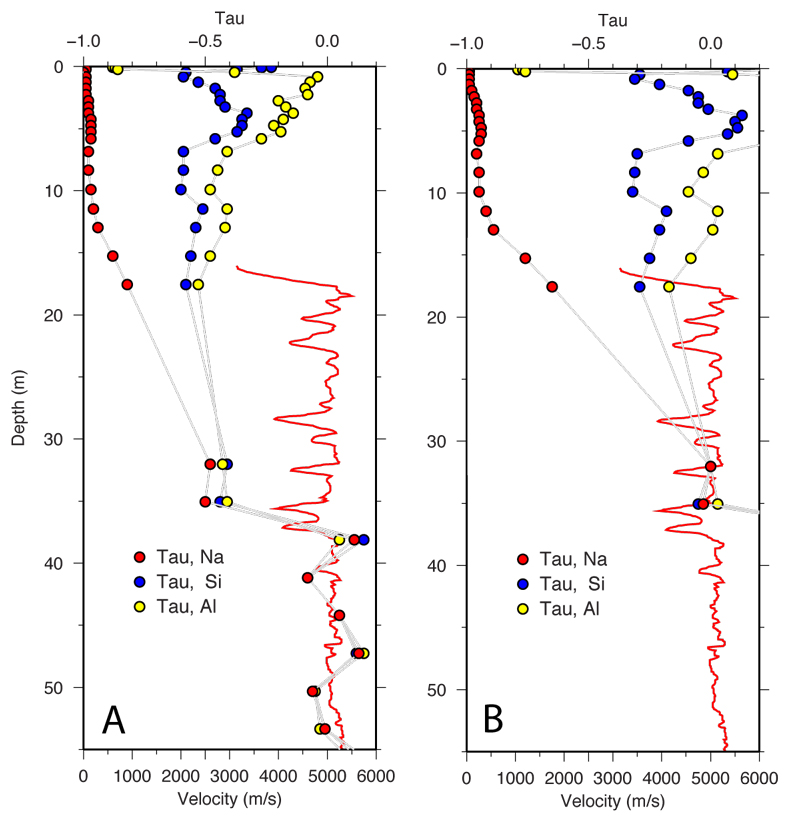
**

**Supplementary Figure S15 | Alternative models of chemical weathering.** Calculated chemical mass loss (tau) values for Na, Si, and Al, assuming protolith is (A) the average composition of samples from 40-53 m depth, or (B) the composition of the sample at 32 m depth. The two models likely bracket the maximum variability expected for the calculated mass losses at this site. Sonic velocity is plotted to show extent of fractured bedrock layer (where sonic velocity markedly fluctuates).

**Supplementary References**

34 St Clair, J. *Geophysical investigations of underplating at the Middle American Trench, weathering in the critical zone, and snow water equivalent in seasonal snow, Retrieved from PQDT Open. (Publication No. 3746853)* Ph.D. thesis, University of Wyoming, (2015).

35 Behroozmand, A. A., Keating, K. & Auken, E. A Review of the Principles and Applications of the NMR Technique for Near-Surface Characterization. *Surv. Geophys.* **36**, 27-85, doi:10.1007/s10712-014-9304-0 (2015).

36 Flinchum, B. A. *et al.* Estimating the water holding capacity of the critical zone using near‐surface geophysics. *Hydrological Processes*, 1-19, DOI: 10.1002/hyp.13260 (2018).

37 Ren, S., Parsekian, A. D., Zhang, Y. & Carr, B. J. Hydraulic Conductivity Calibration of Logging NMR in a Granite Aquifer, Laramie Range, Wyoming. *Ground water*, doi:10.1111/gwat.12798 (2018).

38 Carr, H. Y. & Purcell, E. M. Effects of diffusion on free precession in nuclear magnetic resonance experiments. *Physical Review* **94**, 630-638, doi:10.1103/PhysRev.94.630 (1954).

39 Meiboom, S. & Gill, D. Modified spin-echo method for measuring nuclear relaxation times. *Review of Scientific Instruments* **29**, 688-691, doi:10.1063/1.1716296 (1958).

40 Holbrook, W. S. *et al.* Geophysical constraints on deep weathering and water storage potential in the Southern Sierra Critical Zone Observatory. *Earth Surf. Process. Landf.* **39**, 366-380, doi:10.1002/esp.3502 (2014).

41 Avellaneda, M. Iterated homogenization, differential effective medium theory and applications. *Communications on Pure and Applied Mathematics* **40**, 527-554, doi:10.1002/cpa.3160400502 (1987).

42 Berryman, J. G. Single-scattering approximations for coefficients in Biot equations of poroelasticity. *Journal of the Acoustical Society of America* **91**, 551-571, doi:10.1121/1.402518 (1992).

43 Berryman, J. G., Pride, S. R. & Wang, H. F. A differential scheme for elastic properties of rocks with dry or saturated cracks. *Geophysical Journal International* **151**, 597-611, doi:10.1046/j.1365-246X.2002.01801.x (2002).

44 Cleary, M. P., Chen, I. W. & Lee, S. M. Self-consistent techniques for heterogeneous media. *Journal of the Engineering Mechanics Division-Asce* **106**, 861-887 (1980).

45 Norris, A. N. A differential scheme for the effective moduli of composites. *Mechanics of Materials* **4**, 1-16, doi:10.1016/0167-6636(85)90002-x (1985).

46 Begonha, A. & Braga, S. Weathering of the Oporto granite: geotechnical and physical properties. *Catena* **49**, 57-133 (2002).

47 Novakova, L., Sosna, K., Broz, M., Najser, J. & Novak, P. The matrix porosity and related properties of a leucocratic granite from the Krudum massif, west Bohemia. *Acta Geodynamica Et Geomaterialia* **9**, 521-540 (2012).

48 Sousa, L. M. O., del Rio, L. M. S., Calleja, L., de Argandona, V. G. R. & Rey, A. R. Influence of microfractures and porosity on the physico-mechanical properties and weathering of ornamental granites. *Engineering Geology* **77**, 153-168, doi:10.1016/j.enggeo.2004.10.001 (2005).

49 Goldich, S. S. Determination of ferrous iron in silicate rocks. *Chemical Geology* **42**, 343-347, doi:10.1016/0009-2541(84)90027-5 (1984).

50 White, A. F. *et al.* The effect of temperature on experimental and natural chemical weathering rates of granitoid rocks. *Geochimica et Cosmochimica Acta* **63**, 3277-3291, doi:10.1016/s0016-7037(99)00250-1 (1999).

51 Brimhall, G. H. & Dietrich, W. E. Constitutive mass balance relations between chemical composition, volume, density, porosity, and strain in metasomatic hydrochemical systems - Results on weathering and pedogenesis. *Geochimica et Cosmochimica Acta* **51**, 567-587, doi:10.1016/0016-7037(87)90070-6 (1987).

52 Anderson, S. P., Dietrich, W. E. & Brimhall, G. H. Weathering profiles, mass-balance analysis, and rates of solute loss: Linkages between weathering and erosion in a small, steep catchment. *Geological Society of America Bulletin* **114**, 1143 (2002).
